# Genotype and Associated Cancer Risk in Individuals With Telomere Biology Disorders

**DOI:** 10.1001/jamanetworkopen.2024.50111

**Published:** 2024-12-11

**Authors:** Marena R. Niewisch, Jung Kim, Neelam Giri, Judith C. Lunger, Lisa J. McReynolds, Sharon A. Savage

**Affiliations:** 1 Clinical Genetics Branch, Division of Cancer Epidemiology and Genetics, National Cancer Institute, National Institutes of Health, Bethesda, Maryland; 2 Department of Pediatric Hematology and Oncology, Medical School Hannover, Hannover, Germany; 3 Laboratory of Cell Biology, Center for Cancer Research, National Cancer Institute, National Institutes of Health, Bethesda, Maryland

## Abstract

**Importance:**

Telomere biology disorders (TBDs) are inherited cancer-prone bone marrow failure syndromes with differences in morbidity and mortality based on mode of inheritance.

**Objective:**

To quantify cancer risks in TBDs by genetic subgroups.

**Design, Setting, and Participants:**

This longitudinal cohort study of TBDs assessed cancer occurrences from 2002 through 2022. Participants were individuals with a TBD-associated pathogenic germline variant recruited across institutions by self-referral. Data were collected and analyzed through June 30, 2022.

**Exposures:**

The exposure was TBD genotypes, with subgroups defined by inheritance pattern (autosomal-dominant [AD–non-*TINF2*] vs autosomal-recessive/X-linked [AR/XLR] vs AD-*TINF2*).

**Main Outcomes and Measures:**

The main outcome was cancer; secondary outcomes included death, or organ transplant. Cumulative cancer incidence was determined considering death or transplant as competing events. Observed:expected (O:E) ratios of cancer before and after any organ transplant were calculated using the National Cancer Institute’s Surveillance, Epidemiology, and End Results Program.

**Results:**

Among 230 individuals with TBD (135 [58.7%] male; median [range] age at last follow-up, 34.6 [1.4-82.2] years) included, the risk of cancer was 3-fold higher than the general population (O:E, 3.35 [95% CI, 2.32-4.68]). The highest risk was observed in individuals with AR/XLR (O:E, 19.16 [95% CI, 9.19-35.24]) with a significantly younger cancer onset than in individuals with AD–non-*TINF2* (median [range] age, 36.7 [25.2-53.6] years vs 44.5 [32.2-67.5] years; *P* = .01). The risk of solid tumors was highest in individuals with AR/XLR (O:E = 23.97 [95% CI, 10.96-45.50]), predominantly head and neck squamous cell carcinomas (O:E, 276.00 [95% CI, 75.20-706.67]). Hematologic malignant neoplasm risk was highest in individuals with AD–non-*TINF2* (O:E, 9.41 [95% CI, 4.30-17.86]). Solid tumor cumulative incidence increased to 12% for individuals with AR/XLR by age 45 years and to 13% for individuals with AD–non-*TINF2* by age 70 years. The cumulative incidence of hematologic malignant neoplasms leveled off at 2% by age 30 years and 19% by age 70 years in individuals with AR/XLR and AD–non-*TINF2*, respectively. Individuals with AD-*TINF2* showed the highest cumulative incidence for transplant or death (49% by age 15 years). Following transplant, individuals with AR/XLR (O:E, 136.11 [95% CI, 54.72-280.44) or AD-*TINF2* (O:E, 81.07 [95% CI, 16.72-236.92]) had the highest cancer risk, predominantly young-onset head and neck squamous cell carcinomas (median [range] age, 32.2 [10.5-35.5] years).

**Conclusions and Relevance:**

This cohort study of individuals with TBDs found an increased cancer risk compared with the general population, with the earliest age at onset for individuals with AR/XLR inheritance. Cancer risks increased after organ transplant across all subgroups. These differences in TBD-associated cancer risks by mode of inheritance suggest cancer screening could be tailored by genotype, but additional research is warranted.

## Key Points

**Question:**

Are there differences in cancer risk between genetically defined subgroups of telomere biology disorders (TBDs) compared with the general population?

**Findings:**

This cohort study including 230 individuals with TBDs found a 3-fold increased risk of cancer in individuals with TBDs compared with the general population. Individuals with autosomal-recessive or X-linked TBDs had the highest risk of cancer, at approximately 19-fold in those without prior organ transplant.

**Meaning:**

These findings suggest cancer screening in individuals with TBDs could be tailored by genotype.

## Teaser

This cohort study examines differential cancer risk in individuals with telomere biology disorders by genetic subgroup.

## Introduction

Telomere biology disorders (TBDs) are a spectrum of cancer-prone inherited bone marrow failure syndromes caused by germline pathogenic variants in genes involved in telomere maintenance.^1,2,3,4^ Dyskeratosis congenita, the prototypic TBD, is defined by the mucocutaneous triad of dysplastic nails, oral leukoplakia, and abnormal skin pigmentation. Individuals with TBDs are at very high risk of bone marrow failure, pulmonary fibrosis, liver disease, and many other multisystem medical problems.^1,2,3,4^ Some individuals present in early childhood with multiple complications, whereas others develop isolated features later in life.^3,5^ TBDs are associated with very short telomeres for age and pathogenic/likely pathogenic (P/LP) germline variants in at least 17 different telomere biology genes, including autosomal-dominant (AD; *TERC, NAF1, TINF2, RTEL1, NOP10, NHP2, PARN, ZCCHC8,* and *RPA1*), autosomal-recessive (AR; *CTC1, STN1, WRAP53,* and *DCLRE1B*), and X-linked recessive (XLR; *DKC1*) inheritance patterns.^1,2,3,4,6^ For several genes, both AD and AR inheritance have been reported (*ACD*, *TERT*, *RTEL1*, *PARN*, *NOP10*, *NHP2*, and *POT1*). Heterozygous variants in *TINF2* are primarily de novo, but families with AD inheritance have been reported.^1,3^ AD inheritance *(*AD–non-*TINF2)* is associated with a better overall survival (median overall survival, 64.9 years), while recessive (AR or XLR) and AD-*TINF2* genotypes manifest with more severe clinical phenotypes, leading to significant lower median overall survival (median overall survival, 31.8 and 37.9 years, respectively) compared with AD–non-*TINF2*.^5^

Telomeres, nucleoprotein complexes that protect chromosome ends,^7,8^ shorten with each cell division and on reaching a critically short length, apoptosis or cellular senescence is triggered.^9^ During carcinogenesis, cells overcome critically short telomeres by upregulating telomerase (encoded by *TERT*) using the alternative lengthening of telomeres pathway or other mechanisms.^10^ Long telomeres are considered drivers of cancer risk by promoting cancer cell survival and the accumulation of somatic variations.^10,11,12^ Cancer predisposition with long telomeres (CPLT) is associated with melanoma, thyroid cancer, sarcoma, glioma, and lymphoproliferative neoplasms and caused by rare germline P/LP variants in components of the shelterin telomere protection complex (*POT1*, *TERF2IP*, *ACD*, *TINF2*, and *TERF1)*.^13,14,15,16,17,18,19^ Notably, individuals with CPLT have not been reported to show TBD phenotypes. TBDs are associated with increased risk of certain cancers compared with the general population, which may be due to chromosomal instability caused by short and/or dysfunctional telomeres. The TBD cancer spectrum primarily includes head and neck squamous cell carcinoma (HNSCC), myelodysplastic syndrome (MDS), and acute myeloid leukemia (AML) and is distinct from CPLT.^20,21^ In 2018, risks, defined as observed-to-expected ratios (O:Es), were reported as highest for HNSCC (O:E, 216), MDS (O:E, 578), and AML (O:E, 74).^20^ The same analysis observed higher O:Es for solid tumors following hematopoietic cell transplant (HCT) for patients with TBDs compared with those not undergoing HCT. Another study found O:Es of 61 for oral cavity squamous cell carcinoma (SCC), 21 for AML, and 145 for MDS, not considering transplant status.^21^ Numerous studies have suggested an increased risk of neoplasms following organ transplant in the non-TBD population, but there are limited data on the contribution of organ transplants on cancer risk in TBDs.^20,22,23,24,25^ Experts recommend annual HNSCC screening, but evidence-based guidelines are lacking.^26^

Our genotype-phenotype analyses identified distinct differences in morbidity and mortality between genotype subgroups defined by inheritance patterns.^5^ We identified an 8-fold increased hazard ratio for cancer in TBDs associated with biallelic or XLR pathogenic germline variants.^5^ Further studies focusing on cancer risk by genetic TBD subtypes have not yet been published. Given the significant differences in TBD-related morbidity and mortality by mode of inheritance, we sought to specifically quantify cancer types and risk in genetically defined subgroups of TBDs by analyzing 20 years of data collected from 230 patients.

## Methods

This cohort study was approved by the National Cancer Institute (NCI) institutional review board and registered at ClinicalTrials.gov (identifier: NCT00027274). This study is part of the inherited bone marrow syndromes study, a retrospective and prospective longitudinal cohort study opened in January 2002 that continues to accrue participants and is not a cancer screening study. All participants or their legal guardians sign a written informed consent in accordance with Health and Human Services regulation 45 CFR 46. Clinical information, including self-reported race and ethnicity, is obtained through questionnaires completed by participants, provided medical and pathology records, and self or family member report.

For this analysis, we included all individuals with a confirmed pathogenic germline variant in a known TBD-associated gene by genetic testing or family report (eTable 1 and eFigure 1 in Supplement 1) enrolled between January 1, 2002, and June 1, 2022. A total of 138 individuals with TBD (60.0%) were included in our 2018 cancer study,^20^ and 200 individuals with TBD (87.0%) were included in the 2022 genotype-phenotype analysis.^5^ Individuals with genotype inferred by mode of inheritance based on family history were included. For the TBD cohort, disease-causing inheritance pattern per gene was considered based on published events (eTable 1 and eTable 2 in Supplement 1).^1,3^ TBD carriers were defined as individuals with a monoallelic P/LP variant in a TBD-related gene solely associated with AR inheritance (eg, *WRAP53* or *CTC1*) or female carriers of P/LP variants in the XLR gene *DKC1* (eTable 1 in Supplement 1).

All variants were annotated using SnpEff,^27^ ANNOVAR,^28^ AutoPVS1,^29^ and ClinVar^30^ databases (downloaded June 29, 2022) and classified as P/LP using modified American College of Medical Genetics and Genomics and Association for Molecular Pathology criteria^31^ as previously described.^5^ P/LP criteria for *TERC* included (1) location in the pseudoknot region; (2) absence in gnomAD genome; (3) functional data proving disruption of *TERC* function; (4) TBD-consistent phenotype segregation within a family; (5) presence in unrelated individuals with a consistent phenotype, including publications; and (6) reported P/LP in ClinVar. Exome sequence data were available in a representative subset (54 individuals affected by TBD and 7 TBD carriers) and used to assess whether P/LP variants in other cancer susceptibility genes were present in the cohort.^32,33^ There were pathogenic heterozygous variants in *MUTYH* (4 families) and *FANCA* (1 family) not deemed disease causing.

### Statistical Analysis

Cancer diagnoses were collected from medical records and participant self-report and reviewed through June 30, 2022. Cancer entities quantified included all solid tumors (including in situ diagnoses), hematologic malignant neoplasms, and nonmelanoma skin cancers. The number of solid tumors and their respective subgroups, hematologic malignant neoplasms (lymphomas, leukemia, and AML), and MDS from the NCI TBD cohort was compared with data from NCI’s Surveillance, Epidemiology, and End Results (SEER) Program.^34^ We analyzed cancer occurrence before and after transplant (HCT and/or lung transplant and/or liver transplant) separately without distinguishing cancer occurrences by first or second transplant due to low numbers. As MDS is not a standard SEER category, *International Classification of Diseases for Oncology, 3rd Edition* (*ICD-O-3*) histology codes were used to create a comparison group, and MDS was analyzed separately from other hematologic malignant neoplasms (eTable 3 in Supplement 1). SeerStat version 8.4.0.1 was used to calculate O:Es using the sum of observed diagnoses in the study cohort divided by the expected number based on incidence data of general population in SEER. We calculated 95% CIs using the exact method. For rate data, the SEER Research Data, 8 Registries, Nov 2021 Sub (1975-2019) database was used. The data were adjusted for age, race and ethnicity, and year of cancer diagnosis (eTable 3 in Supplement 1). Race categories were Black, White (including unknown), and other (eg, Asian, Native American or Alaska Native, or multiple). We included data on race and ethnicity because these may influence cancer rates and are part of SEER reporting. Each cancer was counted separately in individuals with multiple cancers.

R software version 4.3.3 (R Project for Statistical Computing) was used for non-SEER analyses. For comparison of median ages between subgroups, we used unpaired Wilcoxon rank test. Survival analysis was conducted using Kaplan-Meier estimates, using log-rank test for comparisons, and cumulative incidence analysis were conducted with the R cuminc function, using computing gray test for comparisons. As TBDs are congenital diseases with a lifetime risk of complications, both survival and cumulative incidence analyses were considered starting at birth, not at time of study enrollment. Cancer-free participants were censored at age at the last follow-up. Competing events considered were death or transplant, solid tumor, or hematologic malignant neoplasms, whichever occurred first. Cohort subgroup comparisons were defined by inheritance pattern (eg, AD–non-*TINF2*, AR/XLR, and AD-*TINF2* separately) as previously described.^5^ We computed rate ratios using the R rateratio function. All statistical tests were 2-sided, and *P* < .05 was deemed significant. In an exploratory analysis, we assessed TBD-associated P/LP germline variant data from 9089 participants in The Cancer Genome Atlas^35^ (TCGA) (eFigure 2 and eTable 4 in Supplement 1).

## Results

### NCI TBD Cohort Characteristics

A total of 230 individuals with TBD (135 [58.7%] male; median [range] age at last follow-up 34.6 [1.4-82.2] years) from 107 unrelated families in the NCI TBD cohort had AD *TERT*, AD *RTEL1*, or XLR *DKC1* as the predominate genotypes (Table 1; eTable 5 in Supplement 1). Individuals with AD-*TINF2*, *DKC1*, or biallelic variants were significantly younger than those with AD–non-*TINF2* disease. The median transplant- and cancer-free survival was 45.5 (95% CI, 40.4-52.1) years overall and was highest for individuals with AD–non-*TINF2*, at 59.8 (95% CI, 53.5-65.6) years, whereas it was only 22.7 (95% CI, 17.1-27.5) years for individuals with AR/XLR and 28.2 (95% CI, 5.2-42.5) years for individuals with *TINF2* disease (eFigure 3 in Supplement 1).

**Table 1.** Characteristics of Study Participants

Characteristic	TBD cohort, No. (%)^a^				Unaffected TBD carriers, No. (%)
	Total	AD–non-*TINF2*	AR/XLR	AD-*TINF2*	
Individuals, No.	230	139	64	27	44
Unrelated individuals, No.	107	42	48	17	23
Affected genes (% of total group)	*TERT* (29.6), *RTEL1* (20.4), *TERC* (15.2), *DKC1* (14.4), *TINF2* (11.7), *PARN* (3.5), *CTC1* (2.6), *ACD* (1.3), *WRAP53* (1.3)	*TERT* (47.5), *TERC* (25.2), *RTEL1* (23), *ACD* (1.4), *PARN* (2.9)	*DKC1* (51.6), *RTEL1* (23.4), *CTC1* (9.4), *PARN* (6.3), *WRAP53* (4.7), *TERT* (3.1), *ACD* (1.6)	*TINF2*	*DKC1* [females] (56.8), *CTC1* (22.7), *WRAP53* (20.5)
Sex					
Male	135 (58.7)	64 (46.0)	54 (84.4)	17 (63.0)	9 (20.5)
Female	95 (41.3)	75 (54.0)	10 (15.6)	10 (37.0)	35 (79.5)
Race and ethnicity					
Asian	5 (2.2)	2 (1.4)	1 (1.6)	2 (7.4)	0
Black or African American	1 (0.4)	1 (0.7)	0	0	0
Multiple	9 (3.9)	6 (4.3)	2 (3.1)	1 (3.7)	0
Native American or Alaska Native	1 (0.4)	1 (0.7)	0	0	0
White	168 (73.1)	102 (73.4)	50 (78.1)	16 (59.3)	30 (68.2)
Unknown	46 (20)	27 (19.4)	11 (17.2)	8 (29.6)	14 (31.8)
Birth year, median (range)	1982 (1909-2014)	1975 (1909-2012)	1993 (1921-2014)	1996 (1937-2014)	1965 (1931-2006)
With TL <first percentile, No./No. with data (%)	113/164 (68.9)	56/102 (54.9)	38/42 (90.5)	19/20 (95.0)	0/26
HCT or lung or liver transplant^b^					
Any	74 (32.2)	28 (20.1)	29 (45.3)	17 (63)	0
Age at first transplant, median (range), y	18.9 (0.9-63.1)	32 (2.2-63.1)	16.9 (0.9-34.7)	5.7 (2.1-47.3)	NA
HCT^c^	66 (89.2)	22 (78.6)	28 (96.6)	16 (94.1)	NA
Age at HCT, median (range), y	16.8 (0.9-63.1)	27.5 (2.2-63.1)	16.8 (0.9-34.7)	5.5 (2.1-47.3)	NA
Lung transplant	9 (12.2)	7 (25)	0	2 (11.8)	NA
Age at lung transplant, median (range), y	50.8 (13.1-62.4)	53.7 (32.5-62.4)	NA	29.3 (13.1-45.5)	NA
Liver transplant	4 (5.4)	1 (3.4)/	3 (10.3)	0	NA
Age at liver transplant, median (range), y	27.8 (21-56.7)	56.7	25.8 (21-29.8)	NA	NA
Deceased at last follow-up	92 (40.0)	34 (24.5)	42 (65.6)	16 (59.3)	2 (4.5)^d^
Deceased due to cancer	12 (13.0)	4 (11.8)	4 (9.5)	4 (25.0)	0
Age last follow-up median (range), y	34.6 (1.4-82.2)	42.1 (2.4-82.2)	21.8 (1.4-54.2)	16.6 (4.6-79.6)	51.6 (7.4-85.3)
Age at last follow-up or transplant, median (range), y^e^	33.8 (0.9-82.2)	42 (2.2-82.2)	19.9 (0.9-54.2)	9.4 (2.1-79.6)	NA
Age at last follow-up after transplant, median (range), y	22.5 (2.2-69)	35.7 (2.4-69)	20.6 (2.2-38)	13.5 (4.6-47.3)	NA
Person-years at risk, No.					
Prior to transplant	7697.6	5750.5	1377.4	569.7	NA
Following transplant	338.3	100.3	137.3	100.6	NA
With cancer					
Prior to transplant	25 (10.9)	16 (11.5)	8 (12.5)	1 (3.7)	NA
Following transplant	8 (10.8)	2 (7.1)	3 (10.3)	3 (17.6)	NA

Abbreviations: AD, autosomal dominant; AD-*TINF2*, autosomal-dominant *TINF2*; AR, autosomal recessive; HCT, hematopoietic cell transplantation; NA, not applicable; TBD, telomere biology disorder; TL, telomere length; XLR, X-linked recessive.

^a^ Included 20 individuals with Hoyeraal Hreidarsson Syndrome, 4 individuals with Revesz syndrome, and 3 individuals with Coats plus. The TBD diagnosis for 24 individuals was established after death (based on family pedigree analysis, genetic report, and/or obligate carrier status).

^b^ Two lung transplants and 2 liver transplants were performed following HCT; 1 simultaneous lung-liver transplant is listed both in lung and liver. One patient received a kidney transplant for chronic kidney disease due to BK virus nephropathy with severe nephrosclerosis 6 years after having received a lung transplant.

^c^ Ten matched sibling donors, 47 matched unrelated donors, 1 unrelated donor (specific information missing), 4 cord blood, 4 information missing.

^d^ Cause of death not reported for either individual.

^e^ Age either at last follow-up or at time of lung, liver, or HCT, whichever event occurred first.

Of 92 deceased individuals across all genotypes, 26 (28.3%) had a prior cancer diagnosis, which was the reported cause of death in 12 of these deaths (46.2%). Death was related to cancer therapy (chemotherapy or HCT) in an additional 4 deaths.

### Risk of Cancer in TBDs Prior to Transplant

Of 230 individuals with TBD, 25 had 34 cancers prior to any transplant (HCT or liver or lung transplant) at a median (range) age of 42.9 (25.2-67.5) years, equating to a 3-fold increased risk compared with the general population (O:E, 3.35 [95% CI, 2.32-4.68]) (Table 2; eTables 6-9 in Supplement 1). Cancers included 5 reported as in situ diagnoses (1 cervical cancer, 4 HNSCCs) (eTable 5 in Supplement 1). Three individuals had 2 or more cancers: 1 female patient (AD *TERC*) had 8 distinct HNSCCs over 22.4 years; 1 male patient (*DKC1*) was diagnosed with anal SCC and HNSCC, and another male patient (*DKC1*) developed esophageal cancer followed by rectal cancer.

**Table 2.** Cancer Risk Prior to Hematopoietic Cell, Lung, or Liver Transplantation

Cancer by site	Age, median (range), y	No.		O:E (95% CI)^a^
		Observed	Expected	
All sites (excluding nonmelanoma skin cancer)				
Overall	42.9 (25.2-67.5)	34	10.14	3.35 (2.32-4.68)^b^
AD	44.5 (32.2-67.5)	23	8.99	2.56 (1.62-3.84)^b^
AR/XLR	36.7 (25.2-53.6)	10	0.52	19.16 (9.19-35.24)^b^
AD-*TINF2*	42.5 (NA)	1	0.64	1.57 (0.04-8.73)
Solid tumor				
Overall	41.4 (25.2-66.4)	23	8.76	2.63 (1.66-3.94)^b^
AR/XLR	37.3 (25.2-53.6)	9	0.38	23.97 (10.96-45.50)^b^
Oral cavity and pharynx (HNSCC)				
Overall	43.1 (25.2-62.8)	14	0.26	54.37 (29.72-91.22)^b^
AD	43.1 (38.4-62.8)	10	0.23	43.12 (20.68-79.29)^b^
AR/XLR	40.2 (25.2-53.6)	4	0.01	276.00 (75.20-706.67)^b^
Tongue				
Overall	43.1 (25.2-62.8)	12	0.08	158.50 (81.90-276.87)^b^
AD	43.1 (38.4-62.8)	10	0.07	145.41 (69.73-267.42)^b^
AR/XLR	39.4 (25.2-53.6)	2	0.00	496.66 (60.15-1794.10)^b^
Digestive system (esophagus, stomach, small intestine, colon, rectum, anus)				
Overall	37.7 (34.0-66.4)	6	1.35	4.45 (1.63-9.69)^b^
AR/XLR	37.3 (34.0-38.8)	5	0.05	94.97 (30.84-221.64)^b^
Esophagus				
Overall	38.1 (35.4-66.4)	3	0.07	44.24 (9.12-129.28)^b^
AR/XLR	36.8 (35.4-38.1)	2	0.00	819.78 (99.28-2961.34)^b^
Rectum, rectosigmoid junction, anus, anal canal and anorectum				
Overall	37.3 (34.0-38.8)	3	0.29	10.23 (2.11-29.89)^b^
AR/XLR	37.3 (34.0-38.8)	3	0.01	243.05 (50.12-710.30)^b^
Breast	62.7 (NA)	1	1.86	0.54 (0.01-3.00)
Female genital system (uterus, cervix, vagina, vulva, others)	43.1 (36.9-49.3)	2	0.80	2.49 (0.30-9.01)
Cervix uteri	36.9 (NA)	1	0.19	5.31 (0.13-29.60)
All lymphatic and hematopoietic diseases (excluding MDS)				
Overall	44.2 (27.5-67.5)	11	1.17	9.30 (4.68-16.80)^b^
AD	44.5 (32.2-67.5)	9	0.96	9.41 (4.30-17.86)^b^
Non-Hodgkin Lymphoma				
Overall	44.5 (32.2-65.6)	5	0.46	10.80 (3.51-25.20)^b^
AD	50.7 (32.2-65.6)	4	0.39	10.19 (2.78-26.10)^b^
AD-*TINF2*	42.5 (NA)	1	0.02	48.53 (1.23-270.38)^b^
Leukemia^c^				
Overall	43.7 (27.5-67.5)	6	0.42	14.30 (5.24-31.10)^b^
AD	44.2 (41.0-67.5)	5	0.32	15.39 (5.00-35.91)^b^
AML				
Overall	44.2 (27.5-67.5)	5	0.10	49.50 (16.07-115.5)^b^
AD	51.2 (41.0-67.5)	4	0.08	48.49 (13.21-124.14)^b^
AR/XLR	27.5 (NA)	1	0.01	88.64 (2.24-493.87)^b^
Myelodysplastic syndrome (prior to HCT, irrespective of lung or liver transplant)				
Overall	51.4 (5.9-66)	17	0.03	529.70 (308.57-848.10)^b^
AD	52.9 (26.6-66.0)	13	0.03	460.76 (245.34-787.91)^b^
AR/XLR	16.8 (5.9-47.5)	4	0.00	4021.15 (1095.63-10 295.73)^b^

Abbreviations: AD, autosomal dominant; AD-*TINF2*, autosomal-dominant *TINF2*; AML, acute myeloid leukemia; AR, autosomal recessive; HCT, hematopoietic cell transplant; HNSCC, head-neck squamous cell carcinoma; MDS, myelodysplastic syndrome; NA, not applicable; O:E, observed over expected ratio; XLR, X-linked recessive.

^a^ O:Es were calculated using the sum of observed diagnosis in the study cohort divided by the expected number based on incidence data of general population.^34^

^b^ Statistically significant O:E. For inheritance pattern subgroups, only significant O:Es are shown.

^c^ One leukemia could not be classified based on the data provided.

The cancer frequency was similar for individuals with AR/XLR and AD–non-*TINF2* TBD (Table 1), but the age at cancer was much younger in individuals with AR/XLR TBD (median [range] age, 36.7 [25.2-53.6] years) vs AD–non-*TINF2* (median [range] age, 44.5 [32.2-67.5] years) (*P* = .004) (Table 2). The O:E was 19.16 (95% CI, 9.19-35.24) for AR TBDs compared with 2.56 (95% CI, 1.62-3.84) for AD–non-*TINF2* disease. The risk of solid tumors was highest in individuals with AR/XLR (O:E, 23.97 [95% CI, 10.96-45.50]), whereas O:Es for hematologic malignant neoplasms (including lymphoma) was higher in individuals with AD–non-*TINF2* (O:E, 9.41 [95% CI, 4.3-17.86]. There was only 1 individual with *TINF2* who had not undergone transplant with a malignant neoplasm; they had a non-Hodgkin lymphoma of the lung.

HNSCC was associated with the highest risk in TBDs, with an overall O:E of 54.37 (95% CI, 29.72-91.22) (AD–non-*TINF2*: O:E, 43.12 [95% CI, 20.68-79.29]; AR/XLR: O:E, 276.00 [95% CI, 75.20-706.67]). Seven individuals (3.0%; 5 male and 2 female) were diagnosed with at least 1 HNSCC at a median (range) age of 43.1 (25.2-62.8) years. This risk was most notable for tongue SCC, with an O:E of 158.50 (95% CI, 81.90-276.87) for all TBDs compared with the general population (AD–non-*TINF2*: O:E, 145.41 [95% CI, 69.73-267.42]; AR/XLR: O:E, 496.66 [95% CI, 60.15-1794.10]). There was a 4-fold increased risk of digestive system cancers, most notable in individuals with AR/XLR, with an 800-fold increased risk of esophagus cancer and 240-fold for anorectal cancer compared with the general population (Table 2). Only 1 patient with HNSCC reported smoking or alcohol use. In 3 individuals with esophageal cancer, 2 reported an extensive smoking history, and data were not available for 1 individual.

We evaluated the adverse events of hematologic malignant neoplasms (including lymphomas and leukemias, excluding MDS) solid tumors, and transplant or death (Figure). Hematologic malignant neoplasms (excluding MDS) and solid tumor cumulative incidences were 12% and 13%, respectively, by the age 70 years for all individuals. The cumulative incidence of hematologic malignant neoplasms leveled off at 2% by age 30 years in individuals with AR/XLR and 19% by age 70 years in individuals with AD–non-*TINF2*. Solid tumor cumulative incidence increased to 12% by age 45 years for individuals with AR/XLR, whereas it was 4% at the same age for individuals with AD–non-*TINF2*, with a later increase to 13% by age 70 years. Individuals with AD-*TINF2* had the highest cumulative incidence for either transplant or death at an early age (49% vs 30% [AR/XLR] and 4% [AD–non-*TINF2*] by age 15 years). Of note, when considering only unrelated individuals (107 individuals), cumulative incidences remained similar (eFigure 4 in Supplement 1); however, analyses were limited by low numbers.

**Figure.**
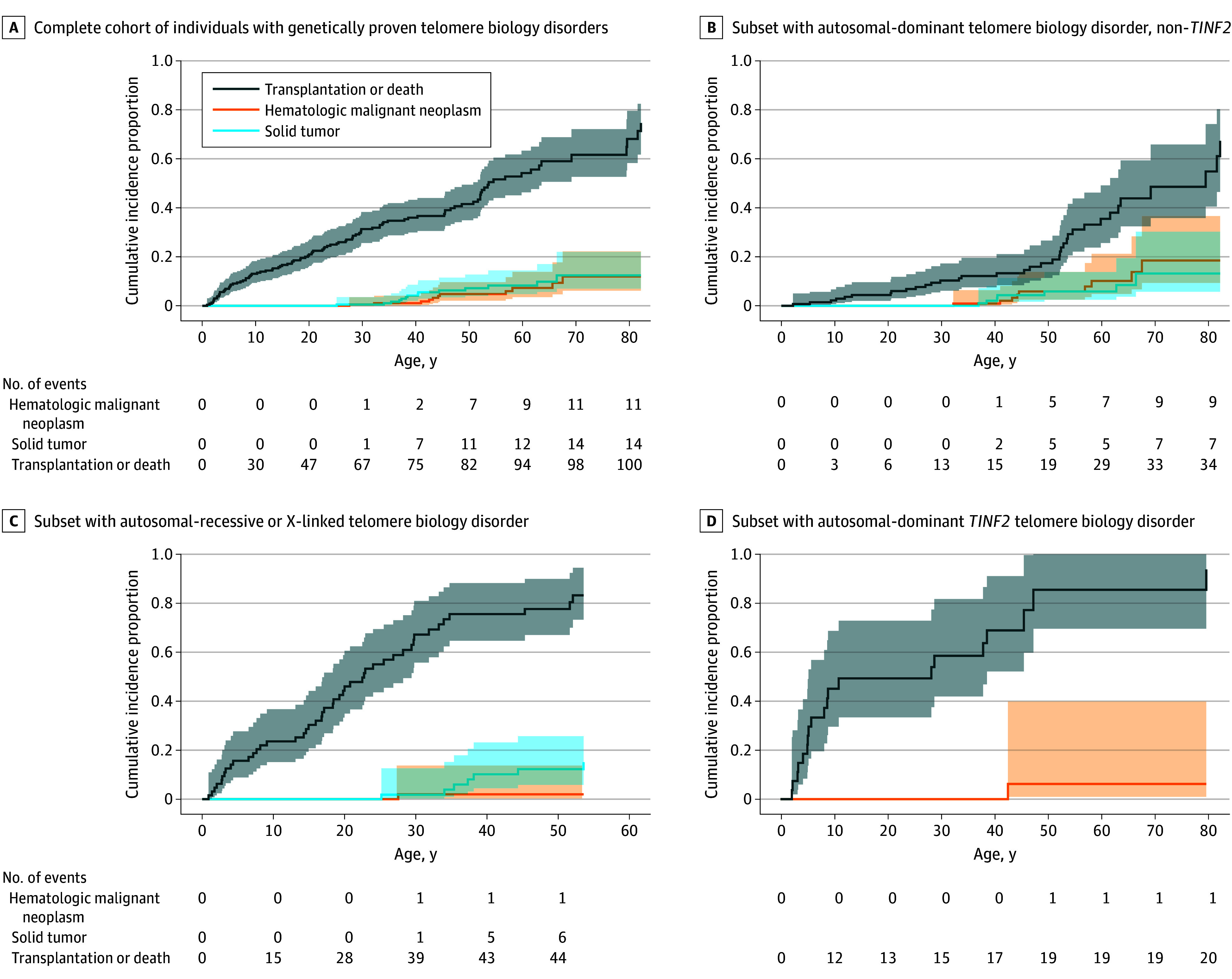
Complications in Patients With Telomere Biology Disorders Who Have Not Undergone Transplantation Cumulative incidences of adverse events (hematologic malignant neoplasm [including lymphomas and leukemias, excluding myelodysplastic syndrome], solid tumors, and transplant or death) by age in patients enrolled in the National Cancer Institute’s Telomere Biology Disorder cohort. Lines indicate cumulative incidence of each event; shading, 95% CIs.

MDS was considered separately from other malignant neoplasms and evaluated solely before HCT. Two individuals with MDS had previously received a lung transplant (2.0 and 7.6 years prior to MDS). The risk of MDS was more than 500 times increased in individuals with TBDs compared with the general population (O:E, 529.70 [95% CI, 308.57-848.10]). For the subgroup of individuals with AR/XLR, the MDS risk was more than 4000-fold.

Nonmelanoma skin cancers (basal cell carcinoma or SCC) were reported by 12 individuals before transplant (7 individuals reported multiple, including in situ skin SCC in 2 individuals), and the median (range) age at first skin cancer was 39.5 (27.1-61.1) years (data missing for 1 individual). As nonmelanoma skin cancer is not included in SEER data, their O:Es could not be calculated.

### Increased Cancer Risk Following HCT or Solid Organ Transplant

Of 230 individuals with TBDs, 74 underwent HCT or lung or liver transplant (Table 1). One individual who underwent lung transplant received a subsequent kidney transplant. The median (range) age was 18.9 (0.9-63.1) years at first transplant and 22.5 (2.2-69.0) years at last contact , resulting in 338.3 posttransplant person-years at risk.

Eight individuals who had undergone transplant were diagnosed with 13 distinct cancers (Table 3; eTables 10-13 in Supplement 1). There was a 25-fold increased risk of any cancer following transplant for individuals with TBDs compared with the SEER population (OE, 25.08 [95% CI, 13.35-42.89]), mostly solid tumors, specifically HNSCC (O:E, 483.88 [95% CI, 208.91-953.44]). Solid tumor occurrence increased drastically following transplant (rate ratio, 10.88 [95% CI, 5.31-22.33]; counting each solid tumor separately in individuals with multiple occurrences).

**Table 3.** Cancer Risk Following Lung, Liver, and/or Hematopoietic Cell Transplantation

Cancer by site	Median (range), y		No.		O:E (95% CI)^a^
	Age	Time since first transplant	Observed	Expected	
All sites (excluding non-melanoma skin cancer)					
Overall	32.7 (10.5-56.5)	3.8 (1.8-14.6)	13	0.52	25.08 (13.35-42.89)^b^
AD	52.4 (51.4-56.49)	2.8 (2.8-3.8)	3	0.43	6.98 (1.44-20.39)^b^
AR/XLR	32.6 (14.9-35.5)	3.5 (1.8-5.8)	7	0.05	136.11 (54.72-280.44)^b^
AD-*TINF2*	18.8 (10.5-32.7)	8.3 (4.6-14.6)	3	0.04	81.07 (16.72-236.92)^b^
All solid tumors					
Overall	32.7 (10.5-52.4)	4.6 (2.2-14.6)	11	0.45	24.57 (12.26-43.96)^b^
AR/XLR	33.7 (22.7-35.5)	4.3 (2.2-5.8)	6	0.04	162.06 (59.47-352.74)^b^
AD-*TINF2*	18.8 (10.5-32.7)	8.3 (4.6-14.6)	3	0.03	105.29 (21.71-307.70)^b^
Oral cavity and pharynx (HNSCC)					
Overall	32.2 (10.5-35.5)	5.2 (2.2-14.6)	8	0.02	483.88 (208.91-953.44)^b^
AR/XLR	33.7 (22.7-35.5)	4.3 (2.2-5.8)	6	0.00	6475.78 (2376.50-14 095.04)^b^
AD-*TINF2*	14.7 (10.5-18.8)	11.4 (8.3-14.6)	2	0.00	3702.91 (448.44-13 376.19)^b^
Tongue					
Overall	18.8 (10.5-22.7)	8.3 (3.5-14.6)	3	0.01	519.95 (107.23-1519.52)^b^
AR/XLR	22.7 (NA)	3.5 (NA)	1	0.00	3586.17 (90.79-19 980.84)^b^
AD-*TINF2*	14.7 (10.5-18.8)	11.4 (8.3-14.6)	2	0.00	12 632.48 (1529.85-45 632.85)^b^
Melanoma of the skin					
Overall	51.9 (51.4-52.4)	3.3 (2.8-3.8)	2	0.04	49.06 (5.94-177.21)^b^
AD	51.9 (51.4-52.4)	3.3 (2.8-3.8)	2	0.03	63.13 (7.65-228.06)^b^
Urinary bladder					
Overall	32.7 (NA)	4.6 (NA)	1	0.01	70.41 (1.78-392.30)^b^
AD-*TINF2*	32.7 (NA)	4.6 (NA)	1	0.00	4752.66 (120.33-26 480.10)^b^
All lymphatic and hematopoietic diseases					
Overall	35.7 (14.9-56.5)	2.3 (1.8-2.8)	2	0.06	33.91 (4.11-122.49)^b^
AR/XLR	14.9 (NA)	1.8 (NA)	1	0.01	75.61 (1.91-421.27)^b^
Hodgkin lymphoma					
Overall	14.9 (NA)	1.8 (NA)	1	0.01	119.61 (3.03-666.42)^b^
AR/XLR	14.9 (NA)	1.8 (NA)	1	0.00	253.81 (6.43-1414.11)^b^
Acute myeloid leukemia					
Overall	56.5 (NA)	2.8 (NA)	1	0.01	196.93 (4.99-1097.20)^b^
AD	56.5 (NA)	2.8 (NA)	1	0.00	320.01 (8.10-1782.99)^b^
PTLD	25.7 (18.5-32.7)	5 (4.6-5.5)	NA	NA	NA

Abbreviations: AD, autosomal dominant; AD-*TINF2*, autosomal-dominant *TINF2*; AR, autosomal recessive; HNSCC, head-neck squamous cell carcinoma; NA, not applicable; O:E, observed over expected ratio; PTLD, posttransplant lymphoproliferative disease; XLR, X-linked recessive.

^a^ O:Es were calculated using the sum of observed diagnosis in the study cohort divided by the expected number based on incidence data of general population in the National Cancer Institute’s Surveillance, Epidemiology, and End Results (SEER) Program.^34^

^b^ Statistically significant O:E. For inheritance pattern subgroups, only significant O:Es are shown.

Following transplant, individuals with AR/XLR had the highest O:E for any cancer (O:E, 136.11 [95% CI, 54.72-280.44]), and the risk was more than 6000-fold for HNSCC, with even higher risks for tongue SCC compared with the general population. Individuals with posttransplant HNSCC were remarkably young at cancer diagnosis (median [range] age, 32.2 [10.5-35.5] years). Of 17 individuals with *TINF2*, 3 (17.6%) had malignant neoplasms after transplant (2 HNSCC and 1 urinary bladder cancer).

Nonmelanoma skin cancers (basal cell carcinoma or SCC) were reported by 6 individuals (8.1%) after transplant, first occurring at a median (range) of 3.9 (1.8-7.5) years after transplant. Of these, 3 individuals reported several skin cancers, including in situ skin SCCs.

Among 44 individuals who died after transplant, the most common causes of death were treatment-related (16 individuals [36.4%]) or pulmonary (12 individuals [27.3%]) complications, including 1 individual diagnosed with AML 2.8 years after lung transplant who died during leukemia induction therapy. Three individuals (6.8%), all with AD-*TINF2*, died due to posttransplant malignant neoplasms.

### Cancer in NCI TBD Carriers

There were 44 clinically unaffected NCI TBD carriers, including 9 with monoallelic *WRAP53,* 10 with monoallelic *CTC1*, and 25 female *DKC1* variant carriers (Table 1). None underwent HCT or lung or liver transplant. The median (range) age at last follow-up was 51.6 (7.4-85.3) years, with only 1 individual with postmenopausal breast cancer. The SEER comparison did not reveal increased O:Es (eTable 14 in Supplement 1).

### Exploratory Analysis: TCGA Participants With Germline P/LP TBD-Associated Gene Variants

We identified 42 germline variants of interest in 58 of 9089 TCGA participants (0.6%) across all cancer entities, including 8 variants present in the NCI’s TBD cohort (4 loss of function and 4 missense affecting either *CTC1*, *RTEL1*, *TERT*, or *WRAP53*) (eTable 5 and eTable 15 in Supplement 1). Of these, 38 were classified as P/LP. Four variants (*TERT* p.R1086H, *NOP10* p.L3fs, *POT1* p.M589fs, *WRAP53* p.G350fs) fulfilled several American College of Medical Genetics and Genomics and Association for Molecular Pathology pathogenicity criteria without formally being P/LP and were designated as variant of unknown significance most likely pathogenic.

Individuals harboring germline P/LP or VUS-P variants in TBD-associated genes were compared with the total TCGA participants by affected organ or cancer entity category. The cancer spectrum affected most organ systems, and no specific clustering of affected genes or variants among cancer entities was noted (eTable 16 in Supplement 1).

## Discussion

This cohort study identified important differences in cancer risk in individuals with TBDs that can impact clinical care. The overall excess risk of any cancer for individuals with TBDs prior to transplant was 3.35-fold higher than in the general population after age adjustment. Notably, individuals with AD–non-*TINF2* TBDs who had not undergone transplant had a 2.56-fold increased risk of cancer, whereas the risk in individuals with AR/XLR disease was 19.16-fold higher than the general population.

The risk of cancer increased substantially after HCT or lung or liver transplant in the entire cohort (O:E, 3.35 prior to transplant vs 25.08 after) and was associated with mode of inheritance. It is important to note that comparisons of pre– vs post–organ transplant outcomes are limited because of the heterogeneity in clinical phenotypes, variability in specific genotypes, and challenges related to adjustment for confounding factors, such as indication and survival bias. Age, the most common cancer risk factor in the general population, may be a relatively minor contributor to cancer risk in the TBDs, given that patients receiving transplant were younger at last follow-up than patients having not or not yet been transplanted (median age, 22.5 and 33.8 years, respectively). Additionally, the age at onset for cancer was higher in the individuals who had not yet undergone transplantation vs those who had (median age, 42.9 vs 32.7 years). In non-TBD cohorts, malignant neoplasm risk after solid organ transplant increases to 2-fold higher than the general population and by 4- to 12-fold after HCT.^36,37,38,39,40,41^ It is not known whether transplant and/or immunosuppression affect the natural history of TBD phenotypes, and additional longitudinal studies are required.

The most frequent solid malignant neoplasms in our cohort, HNSCC and esophageal, are associated with smoking and alcohol use in the general population.^42^ Notably, most individuals with TBD (70%) with these malignant neoplasms did not report smoking or significant alcohol use. This finding is similar to a 2023 study of a clinically defined TBD cohort (median age 50 years, vs 34 years in our cohort) that found a specifically high risk of HNSCC in males with *DKC1* P/LP variants.^43^ In the general population, HNSCC is frequently associated with human papillomavirus (HPV) infection, but this does not appear to be the case in individuals with TBDs.^44^ It was previously suggested that individuals with TBDs have a lower-than-expected number of common cancers, such as lung or colorectal cancer.^43^ Common adult-onset solid malignant neoplasms (eg, lung, colorectal, or breast) were also not present in our cohort, but given the complexities of TBD phenotypes and competing risks of morbidity and mortality (eg, bone marrow failure or pulmonary fibrosis), it is not possible to statistically address the risk (or proposed lack thereof) of other common malignant neoplasms in TBDs at this time.

Reassuringly, individuals harboring a heterozygous variant in a predominately AR gene, *CTC1* or *WRAP53*, and including *DKC1* (females), were not at increased risk of cancer. Larger, population-based studies are warranted to fully understand whether there are clinically significant manifestations in these individuals.

We used TCGA germline variant data to explore patterns or types of cancer not yet defined in individuals with germline P/LP TBD gene variants. While there were likely undiagnosed individuals with TBDs in TCGA, hematologic malignant neoplasms and HNSCC were not increased in those individuals. Variable disease penetrance and expressivity are frequent in TBDs.^3,45^ It is possible that individuals with a potential underlying TBD in TCGA represent a later-onset phenotype, although it is not possible to determine this with TCGA data.

The carcinogenic mechanisms in TBDs are not well understood but likely are related to accumulation of DNA damage and dysfunctional telomeres.^19,46^ Telomere length in the general population is a fine balance, with long telomeres being associated with certain cancers and short telomeres with cardiovascular and inflammatory diseases.^7,19^ Individuals with TBDs have very short and dysfunctional telomeres and develop cancer at much younger ages,^20^ but the specific mechanisms by which cancers in individuals with TBDs maintain telomeres is unknown.

### Strengths and Limitations

Strengths of this study include a larger sample size and longer follow-up than prior reports and analyses by mode of inheritance and organ transplant status. This allowed us to fine tune to overall cancer risk estimate to 3.35 from our prior estimates of 4.2^20^ and 11.^47^ This study is limited by its relatively small size (although large for TBDs), predominantly White population, and possible biased ascertainment towards individuals with more medical complications, including cancer, who may be more likely to enroll in a research study. Population-based studies are needed to determine the impact of social determinants and diverse racial and ethnic background on cancer risk in the context of TBDs. We also acknowledge the lack of data on individuals with more recently discovered or suspected TBD genes (eg, *DCLRE1B*, *RPA1*, *MDM4*, *NPM1*, *TYMS*, *POLA1*)^2^ and likely over reporting of individuals with MDS, given MDS diagnostic challenges and inability to conduct central pathology review.

## Conclusions

This cohort study found statistically significant differences in TBD-associated cancer risks based on the mode of inheritance and further refined these risk estimates by organ transplant status. The O:Es resulting from comparison of cancer in individuals with TBDs with SEER population data were refined from prior studies, but caution is warranted in interpretation at the individual-patient level due to ascertainment bias intrinsic to most cohort studies. Future studies of population cohorts with linked clinical data and across all age groups, races, and ethnicities may allow for more robust statistical estimates of cancer risk.

Based on our data, TBDs due to AR/XLR inheritance or AD-*TINF2* variants were associated with highest risk of early-onset HNSCC, esophageal, hematologic, and other cancers. Individuals with AD–non-*TINF2* TBDs were also at increased risk compared with the general population, but the risks were not as high as for other TBDs. Cancer risks increased substantially after organ transplant in all groups. This study illustrates the importance of large, long-term rare disease cohorts to refine disease risks and provides a foundation on which to develop evidence-based cancer screening modalities in the TBDs.

## Notes

Supplement 1. **eTable 1.** Inclusion Criteria for the National Cancer Institute (NCI) Telomere Biology Disorder (TBD) and Carrier Cohorts **eTable 2.** Included Genes and Transcripts for Variant Calling in The Cancer Genome Atlas (TCGA) **eTable 3.** Analysis Details on the Surveillance, Epidemiology, and End Results (SEER) Program Analysis of Cancer Occurrences in the NCI’s TBD Cohort **eTable 4.** TCGA Exploratory Analysis: Cancer Types and Number of Cancer Cases Analyzed for Germline Variants in Telomere Biology Genes **eTable 5.** Rare and Likely Pathogenic Germline Variants in Genes Associated With TBD in the NCI’s Inherited Bone Marrow Failure Syndromes Study and TCGA **eTable 6.** NCI’s TBD Cohort Pretransplant Cancer Data Comparison With SEER Program **eTable 7.** NCI’s TBD Cohort Pretransplant Cancer Data Comparison With SEER Program for the Autosomal Dominant Subgroup **eTable 8.** NCI’s TBD Cohort Pretransplant Cancer Data Comparison With SEER Program for the Autosomal Recessive/X-Linked Subgroup **eTable 9.** NCI’s TBD Cohort Pretransplant Cancer Data Comparison With SEER Program for the *TINF2* Subgroup **eTable 10.** NCI’s TBD Cohort Posttransplant Cancer Data Comparison With SEER Program **eTable 11.** NCI’s TBD Cohort Posttransplant Cancer Data Comparison With SEER Program for the Autosomal Dominant Subgroup **eTable 12.** NCI’s TBD Cohort Posttransplant Cancer Data Comparison With SEER Program for the Autosomal Recessive/X-Linked Subgroup **eTable 13.** NCI’s TBD Cohort Posttransplant Cancer Data Comparison With SEER Program for the *TINF2* Subgroup **eTable 14.** NCI’s TBD Carrier Cohort Comparison With SEER Program **eTable 15.** TCGA: Characteristics of Cancer Cases With Pathogenic/Likely Pathogenic in TBD-Associated Genes **eTable 16.** TCGA: Cancer Cases With Pathogenic/Likely Pathogenic TBD-Associated Variants Per Cancer Category **eFigure 1.** Overview of Study Cohort Selection Within the NCI’s Inherited Bone Marrow Failure Syndromes Study **eFigure 2.** Overview of Germline Variant Curation and Case Selection Within TCGA Dataset **eFigure 3.** Transplant and Cancer-Free Survival in the TBD Cohort Using Kaplan-Meier Estimates **eFigure 4.** Complications in Unrelated, Nontransplanted TBD Individuals
eReferences

Supplement 2.
Data Sharing Statement
