# Supplementary material for: Genotype and Associated Cancer Risk in Individuals With Telomere Biology Disorders
Source: JAMA Netw Open. 2024 Dec 11;7(12):e2450111. doi: 10.1001/jamanetworkopen.2024.50111 (PMC11635530; doi:10.1001/jamanetworkopen.2024.50111)
Supplement: Supplement 1. — eTable 1. Inclusion Criteria for the National Cancer Institute (NCI) Telomere Biology Disorder (TBD) and Carrier Cohorts eTable 2. Included Genes and Transcripts for Variant Calling in The Cancer Genome Atlas (TCGA) eTable 3. Analysis Details on the Surveillance, Epidemiology, and End Results (SEER) Program Analysis of Cancer Occurrences in the NCI’s TBD Cohort eTable 4. TCGA Exploratory Analysis: Cancer Types and Number of Cancer Cases Analyzed for Germline Variants in Telomere Biology Genes eTable 5. Rare and Likely Pathogenic Germline Variants in Genes Associated With TBD in the NCI’s Inherited Bone Marrow Failure Syndromes Study and TCGA eTable 6. NCI’s TBD Cohort Pretransplant Cancer Data Comparison With SEER Program eTable 7. NCI’s TBD Cohort Pretransplant Cancer Data Comparison With SEER Program for the Autosomal Dominant Subgroup eTable 8. NCI’s TBD Cohort Pretransplant Cancer Data Comparison With SEER Program for the Autosomal Recessive/X-Linked Subgroup eTable 9. NCI’s TBD Cohort Pretransplant Cancer Data Comparison With SEER Program for the TINF2 Subgroup eTable 10. NCI’s TBD Cohort Posttransplant Cancer Data Comparison With SEER Program eTable 11. NCI’s TBD Cohort Posttransplant Cancer Data Comparison With SEER Program for the Autosomal Dominant Subgroup eTable 12. NCI’s TBD Cohort Posttransplant Cancer Data Comparison With SEER Program for the Autosomal Recessive/X-Linked Subgroup eTable 13. NCI’s TBD Cohort Posttransplant Cancer Data Comparison With SEER Program for the TINF2 Subgroup eTable 14. NCI’s TBD Carrier Cohort Comparison With SEER Program eTable 15. TCGA: Characteristics of Cancer Cases With Pathogenic/Likely Pathogenic in TBD-Associated Genes eTable 16. TCGA: Cancer Cases With Pathogenic/Likely Pathogenic TBD-Associated Variants Per Cancer Category eFigure 1. Overview of Study Cohort Selection Within the NCI’s Inherited Bone Marrow Failure Syndromes Study eFigure 2. Overview of Germline Variant Curation and Case Selection Within TCGA Dataset [file jamanetwopen-e2450111-s001.pdf]

Supplemental Online Content

Niewisch MR, Kim J, Giri N, Lunger JC, McReynolds LJ, Savage SA. Genotype and associated cancer risk in individuals with telomere biology disorders. *JAMA Netw Open*. 2024;7(12):e2450111. doi:10.1001/jamanetworkopen.2024.50111

eTable 1. Inclusion Criteria for the National Cancer Institute (NCI) Telomere Biology Disorder (TBD) and Carrier Cohorts
eTable 2. Included Genes and Transcripts for Variant Calling in The Cancer Genome Atlas (TCGA)
eTable 3. Analysis Details on the Surveillance, Epidemiology, and End Results (SEER) Program Analysis of Cancer Occurrences in the NCI's TBD Cohort
eTable 4. TCGA Exploratory Analysis: Cancer Types and Number of Cancer Cases Analyzed for Germline Variants in Telomere Biology Genes
eTable 5. Rare and Likely Pathogenic Germline Variants in Genes Associated With TBD in the NCI's Inherited Bone Marrow Failure Syndromes Study and TCGA
eTable 6. NCI's TBD Cohort Pretransplant Cancer Data Comparison With SEER Program
eTable 7. NCI's TBD Cohort Pretransplant Cancer Data Comparison With SEER Program for the Autosomal Dominant Subgroup
eTable 8. NCI's TBD Cohort Pretransplant Cancer Data Comparison With SEER Program for the Autosomal Recessive/X-Linked Subgroup
eTable 9. NCI's TBD Cohort Pretransplant Cancer Data Comparison With SEER Program for the TINF2 Subgroup
eTable 10. NCI's TBD Cohort Posttransplant Cancer Data Comparison With SEER Program
eTable 11. NCI's TBD Cohort Posttransplant Cancer Data Comparison With SEER Program for the Autosomal Dominant Subgroup
eTable 12. NCI's TBD Cohort Posttransplant Cancer Data Comparison With SEER Program for the Autosomal Recessive/X-Linked Subgroup
eTable 13. NCI's TBD Cohort Posttransplant Cancer Data Comparison With SEER Program for the TINF2 Subgroup
eTable 14. NCI's TBD Carrier Cohort Comparison With SEER Program
eTable 15. TCGA: Characteristics of Cancer Cases With Pathogenic/Likely Pathogenic in TBD-Associated Genes
eTable 16. TCGA: Cancer Cases With Pathogenic/Likely Pathogenic TBD-Associated Variants Per Cancer Category
eFigure 1. Overview of Study Cohort Selection Within the NCI's Inherited Bone Marrow Failure Syndromes Study
eFigure 2. Overview of Germline Variant Curation and Case Selection Within TCGA Dataset
eFigure 3. Transplant and Cancer-Free Survival in the TBD Cohort Using Kaplan-Meier Estimates
eFigure 4. Complications in Unrelated, Nontransplanted TBD Individuals
eReferences

This supplemental material has been provided by the authors to give readers additional information about their work.

eTable 1: Inclusion criteria for the National Cancer Institute Telomere Biology Disorder and Carrier Cohorts in this report

**Telomere biology disorder (TBD) affected individuals**	**Patients**
(1) Individuals with a documented pathogenic or likely pathogenic variant by medical or personal report in a TBD-associated gene.	205[a,b]
(2) Clinical carriers: Family members with mucocutaneous triad features (leukoplakia, nail dysplasia, lacy skin pigmentation) and/or pulmonary fibrosis and/or bone marrow failure (with or without other TBD related features) with a documented pathogenic variant (CLIA laboratory or research report) in a family member and genotype inferred by mode of inheritance and reported clinical information.	25
**Assumed healthy carriers of pathogenic telomere biology disorder associated germline variants**	
Individuals with heterozygous variants in *WRAP53* or *CTC1* and female *DKC1* carriers[c]	44[a]

[a]In 7 TBD affected individuals and 2 *DKC1* female carriers information on the specific variant was not available.

[b]Includes 26 individuals with heterozygous pathogenic variants, which were detected through a family index case having autosomal recessive disease. All of the affected genes in these 26 individuals had previously been implicated in autosomal dominant disease according to OMIM (https://www.omim.org/) and literature review.

[c]To date, *WRAP53* and *CTC1* have only been associated with AR disease according to OMIM (https://www.omim.org/) and literature review.

<u>eTable 2:</u> Included genes and transcripts (Genome Assembly: GRCh38) for variant calling in The Cancer Genome Atlas

Gene	Chromosome	NCBI transcript (Refseq, MANE select)	Ensemble transcript (canonical)	Telomere Biology Disorder – associated inheritance pattern(s)[a]
DKC1	chrX	NM_001363.5	ENST00000369550.10	XLR
TERC	chr3	NR_001566.1	ENST00000602385.1	AD, AR
NAF1	chr4	NM_138386.3	ENST00000274054.3	AD
TERT	chr5	NM_198253.3	ENST00000310581.10	AD, AR
NHP2	chr5	NM_017838.4	ENST00000274606.8	AD, AR
POT1	chr7	NM_015450.3	ENST00000357628.8	AR, AD[b]
STN1	chr10	NM_024928.5	ENST00000224950.8	AR
ZCCHC8	chr12	NM_017612.5	ENST00000633063.3	AD
TINF2	chr14	NM_001099274.3	ENST00000267415.12	AD
NOP10	chr15	NM_018648.4	ENST00000328848.6	AD, AR
PARN	chr16	NM_002582.4	ENST00000437198.7	AD, AR
ACD	chr16	NM_001082486.2	ENST00000620761.6	AD, AR
WRAP53	chr17	NM_001143992.2	ENST00000396463.7	AR
CTC1	chr17	NM_025099.6	ENST00000651323.1	AR
RTEL1	chr20	NM_001283009.2	ENST00000360203.11	AD, AR

[a]According to Revy et al. 2023[1].
[b]To date there is one report of a monoallelic *POT1* variant in a family with TBD and variably short telomere length[2].

eTable 3: Analysis details on the Surveillance, Epidemiology, and End Results (SEER) Program analysis of cancer occurrences in the National Cancer Institute's Telomere Biology Disorder Cohort (n=230)

SEER version	SEER 9 Registry; https://seer.cancer.gov
Statistical software	SeerStat version 8.4.0.1 (released May 16, 2022)
Database	SEER Research Data, 8 Registries, Nov 2021 Sub (1975-2019)
Considered categories (outcomes)	• Cancer diagnoses : SEER standard categories (https://seer.cancer.gov/siterecode/icdo3_dwhoheme/index.html)<br>• Myelodysplastic syndrome: defined by ICD-O-3 histology codes: 9980, 9982-9983, 9985-9986, 9989, and 9991-9992
Considered status	• Pre-transplant (hematopoietic cell transplant, lung and/or liver transplant)<br>• Post-transplant (hematopoietic cell transplant, lung and/or liver transplant)
Considered subgroups	• Autosomal dominant non-*TINF2*<br>• Autosomal recessive or X-linked recessive<br>• *TINF2*
Observed over expected (O/E) ratios	• Sum of observed diagnosis in the NCI TBD cohort divided by the expected number based on incidence data of general population in SEER.<br>• Analysis parameters: Using SEER Research Data, 8 Registries, Nov 2021 Sub (1975-2019) rate data. Following from date of entry (birth) to exit (defined per cohort), there is no latency exclusion being used. SEER*Stat takes month and year as input and sets dates to the 15th, adjusting birth to fit the ages of diagnosis and follow-up.<br>• Performing a multiple SIR analysis, using all SEER Recode events (each cancer in individuals with several cancers counted separately).<br>• Exact confidence limit documentation from the SEER*Stat program
Rate adjustments/matching	• Sex<br>• Race (White, Black, Other). The rates are provided for producing "All races combined" statistics. Since populations were not available for unknown race, these cases are grouped with "White".<br>• Five-year age groups (*e.g.,* 0-4 until 85+ years)<br>• Year of diagnosis in five-year groups (*e.g.,* 1975-79 until 2015-2019). For diagnoses outside of the 1975-2019 range: for 1909-1974 the 1975-79 and for diagnoses 2020-2022 the 2015-19 corresponding rate was applied.

eTable 4: **The Cancer Genome Atlas (TCGA) exploratory analysis - cancer types and number of cancer cases analyzed for germline variants in telomere biology genes (September 27, 2022, release #12)**

Cancer Type[a]	Abbreviation	Number of cases analyzed	Cancer Type‡	Abbreviation	Number of cases analyzed
Adrenocortical carcinoma	ACC	87	Lung squamous cell carcinoma	LUSC	314
Bladder urothelial carcinoma	BLCA	392	Mesothelioma	MESO	82
Breast invasive carcinoma	BRCA	960	Ovarian serous cystadenocarcinoma	OV	349
Cervical squamous cell carcinoma and endocervical adenocarcinoma	CESC	300	Pancreatic adenocarcinoma	PAAD	153
Cholangiocarcinoma	CHOL	44	Pheochromocytoma and paraganglioma	PCPG	177
Colon adenocarcinoma	COAD	392	Prostate adenocarcinoma	PRAD	440
Lymphoid neoplasm diffuse large B-cell lymphoma	DLBC	37	Rectum adenocarcinoma	READ	153
Esophageal carcinoma	ESCA	126	Sarcoma	SARC	237
Glioblastoma multiforme	GBM	394	Skin, cutaneous melanoma	SKCM	469
Head and neck squamous cell carcinoma	HNSCC	509	Stomach adenocarcinoma	STAD	396
Kidney chromophobe	KICH	9	Testicular germ cell tumors	TGCT	150
Kidney renal clear cell carcinoma	KIRC	91	Thyroid carcinoma	THCA	431
Kidney renal papillary cell carcinoma	KIRP	232	Thymoma	THYM	112
Acute myeloid leukemia	LAML	149	Uterine carcinosarcoma	UCEC	519
Brain lower grade glioma	LGG	513	Uterine corpus endometrial carcinoma	UCS	51
Liver hepatocellular carcinoma	LIHC	325	Uveal melanoma	UVM	80
Lung adenocarcinoma	LUAD	416	Total		9089

[a] Complete list of included cancer types in the The Cancer Genome Atlas database[3] see https://www.cancer.gov/about-nci/organization/ccg/research/structural-genomics/tcga/studied-cancers.

<u>eTable 5:</u> Rare and likely pathogenic germline variants in genes associated with telomere biology disorders in the National Cancer Institute's Inherited Bone Marrow Failure Syndromes Study and The Cancer Genome Atlas. Variants were classified by ACMG/AMP criteria[b]

**National Cancer Institute Telomere Biology Disorder affected cohort (n=230)**		**The Cancer Genome Atlas dataset**	**Variant**					**ACMG/AMP classification**	
**No. of individuals**	**Reported cancer cases (No. of Individuals)[a]**	**Affected cancer entities (No. of cases)**	**Gene**	**chr**	**Transcript ID RefSeq**	**gDNA change**	**AA change**	**Class**	**Applied criteria**
4	HNSCC *in situ* (2), HNSCC (1), AML(1)	NA	*TERC*	3	NR_001566.1	n.100T>A	-	P	NA
1	none	NA	*TERC*	3	NR_001566.1	n.110_113del	-	P	NA
9	none	NA	*TERC*	3	NR_001566.1	n.114_115del	-	P	NA
5	none	NA	*TERC*	3	NR_001566.1	n.334_339dup	-	LP	NA
4	AML (1)	NA	*TERC*	3	NR_001566.1	n.357_365del	-	LP	NA
1	HNSCC (1)	NA	*TERC*	3	NR_001566.1	n.381G>A	-	P	NA
3	none	NA	*TERC*	3	NR_001566.1	n.413_417del	-	LP	NA
1	none	NA	*TERC*	3	NR_001566.1	n.54_57del		P	NA
3	none	NA	*TERC*	3	NR_001566.1	n.56_58del	-	P	NA
1	none	NA	*TERC*	3	NR_001566.1	n.56_62del	-	P	NA
3	Cervix carcinoma *in situ* (1)	NA	*TERC*	3	NR_001566.1	n.97_98del	-	P	NA
NA	NA	UCEC (1)	*NAF1*	4	NM_138386.3	c.985delinsAT	p.S329fs	LP	PVS1_strong, PM2
5	Melanoma (post lung transplant, 1), NHL (1)	NA	*TERT*	5	NM_198253.3	c.1892G>A	p.R631Q	LP	PS1_moderate, PS4_moderate, PM2, PM1_supporting, PS3_supporting, PP2, PP3, PP4

© 2024 Niewisch MR et al. *JAMA Network Open.*

2	None	NA	*TERT*	5	NM_198253.3	c.1990G>A	p.V664M	LP	PM1, PM2, PM3, PM5_supporting, PP2
5	None	NA	*TERT*	5	NM_198253.3	c.2110C>T	p. P704S	LP	PS1_moderate, PS3_moderate, PS4_moderate, PM1, PM2, PP2, PP3, PS3, PP4
4	NHL (1), leukemia (1)	NA	*TERT*	5	NM_198253.3	c.2240del	p.V747fs	P	PVS1, PS1_supporting, PS4_supporting, PM2, PP1, PP4
1	None	NA	*TERT*	5	NM_198253.3	c.2318T>C	p.M773T	LP	PS4_supporting, PM1, PM2, PP2, PP3, PP4
1	None	NA	*TERT*	5	NM_198253.3	c.2455C>T	p.R819C	LP	PS1_supporting, PS4_supporting, PM2, PM1, PP2, PP4
3	AML (post lung transplant, 1)	NA	*TERT*	5	NM_198253.3	c.2575C>T	p.R859W	LP	PM1, PM2, PP2, PP4, BP4
3	Endometrioid adenocarcinoma (1)	NA	*TERT*	5	NM_198253.3	c.258G>C	p.Q86H	LP	PM1, PM2, PP2, PP3, PP4
11	AML (1), NHL (1)	NA	*TERT*	5	NM_198253.3	c.2591T>C	p.L864P	LP	PM2, PM1, PP1, PP2, PP3, PP4
2	None	NA	*TERT*	5	NM_198253.3	c.2593C>T	p.R865C	LP	PM1, PM2, PM5, PP2, PP3, PP4
3	NHL (1)	NA	*TERT*	5	NM_198253.3	c.2603A>G	p.D868G	LP	PM2, PM1, PP2, PP3, PS1_supporting, PP4, PP1
1	None	NA	*TERT*	5	NM_198253.3	c.2638G>A	p.A880T	LP	PS3_supporting, PM1_supporting, PM2, PP2, PP3, PP4

2	none	NA	*TERT*	5	NM_198253.3	c.2768C>T	p.P923L	LP	PS4_moderate, PM1, PM2, PP1, PP2, PP3, PP4
4	none	NA	*TERT*	5	NM_198253.3	c.2935C>T	p. R979W	LP	PS3_supporting, PS4_moderate, PM1, PM2, PP2, PP3, PP4, PP1
2	none	NA	*TERT*	5	NM_198253.3	c.2947C>T	p.H983Y	LP	PS4_moderate, PM1, PM2, PP2, PP4
4	none	NA	*TERT*	5	NM_198253.3	c.3150G>C	p.K1050N	LP	PS4_moderate, PM1_supporting, PM3_supporting, PP2, PP3, PP4
2	none	NA	*TERT*	5	NM_198253.3	c.320_328del	p.A107_G109del	LP	PM1, PM2, PM4, PP1, PP4
5	none	NA	*TERT*	5	NM_198253.3	c.3205G>A	p.A1069T	LP	PS4_supporting, PM1, PM2, PP1, PP2, PP3, PP4
1	none	COAD (1), LUSC (1), SKCM (1)	*TERT*	5	NM_198253.3	c.3257G>A	p.R1086H	VUS-P	PS4_supporting, PM1, PM2_Supporting, PP2, PP4.
1	none	NA	*TERT*	5	NM_198253.3	c.416T>G	p.L139R	LP	PS4_supporting, PM1, PM2, PP2, PP3, PP4
1	none	NA	*TERT*	5	NM_198253.3	c.1156_1171del	p.Y386fs	P	PVS1, PM2, PS1_supporting, PP2, PP4
5	none	NA	*TERT*	5	NM_198253.3	1.4 Mb deletion at 5p15.33	NA	NA	NA
NA	NA	COAD (1)	*POT1*	7	NM_015450.3	c.1072delinsTC	p.Q358fs	P	PVS1_very strong, PS1_moderate, PM2_supportive

NA	NA	BRCA (1), LGG (1), LIHC (1), PRAD (1)	*POT1*	7	NM_015450.3	c.1087C>T	p.R363*[3]	P	PVS1_very strong. PS1_moderate, PM2, PM5_supporting.
NA	NA	LAML (1)	*POT1*	7	NM_015450.3	c.1164-1G>A	Splice site	P	PVS1_very strong, PS1_moderate, PM2
NA	NA	SARC (1)	*POT1*	7	NM_015450.3	c.1294C>T	p.R432*	P	PVS1_very strong, PS1_supportive, PM2
NA	NA	THCA (1)	*POT1*	7	NM_015450.3	c.1765_1767delinsG	p.M589fs	VUS-P	PVS1_moderate, PM2
NA	NA	UVM (1)	*POT1*	7	NM_015450.3	c.259delinsTC	p.Q87fs	P	PVS1_very strong, PS1_supportive, PM2
NA	NA	ESCA (1)	*POT1*	7	NM_015450.3	c.991C>T	p.Q331*	P	PVS1_very strong, PS1_moderate, PM2_moderate
NA	NA	MESO (1)	*STN1*	10	NM_024928.5	c.354_359delinsT	p.E118fs	LP	PVS1_very strong, PM2_moderate
NA	NA	STAD (1)	*STN1*	10	NM_024928.5	c.876+2T>G	Splice site	LP	PVS1_very strong, PM2_moderate, PP3_supporting
NA	NA	THYM (1)	*STN1*	10	NM_024928.5	c.877-1G>A	Splice site	LP	PVS1_very strong, PM2 moderate
NA	NA	THCA (1), UCEC (1)	*STN1*	10	NM_024928.5	c.C397C>T	p.R133*	P	PVS1_very strong, PS4 supporting, PM2_supporting

NA	NA	LGG (1)	*TINF2*	14	NM_001099274.3	c.1011delinsGA	p.R338fs	LP	PVS1, PM2
NA	NA	PRAD (1)	*TINF2*	14	NM_001099274.3	c.584delinsGA	p.D195fs	LP	PVS1, PM2
1	HNSCC (post-HCT, 1)	NA	*TINF2*	14	NM_001099274.3	c.815G>A	p.W272*	P	PVS1, PS1_supporting, PM2, PM6_supporting, PP4
1	none	NA	*TINF2*	14	NM_001099274.3	c.830del	p.G277fs	P	PVS1, PM2, PP4
9	Bladder carcinoma (post-HCT, 1), NHL (1)	NA	*TINF2*	14	NM_001099274.3	c.838A>G	p. K280E	LP	PS3_supporting, PM1, PM2, PP1, PP3, PP4
1	None	NA	*TINF2*	14	NM_001099274.3	c.844C>A	p. R282S	P	PS4, PS1_supporting, PM1, PM2, PM5, PM6_supporting, PP1, PP3, PP4
5	None	NA	*TINF2*	14	NM_001099274.3	c.845G>A	p. R282H	P	PS1_moderate, PS3_moderate, PS4, PM1, PM2, PM5, PM6_supporting, PP1, PP3, PP4
1	None	NA	*TINF2*	14	NM_001099274.3	c.847C>T	p.P283S	LP	PS1_supporting, PS4_moderate, PM1, PM2, PP2, PP3, PP4
1	HNSCC (post both HCT + lung transplant, 1)	NA	*TINF2*	14	NM_001099274.3	c.851_852del	p.T284fs	P	PVS1, PM1, PM2, PM6, PP4
1	None	NA	*TINF2*	14	NM_001099274.3	c.860T>C	p.L287P	LP	PS4_supporting, PS1_moderate, PM1, PM2, PP3, PP4

3	None	NA	*TINF2*	14	NM_001099274.3	c.873G>C	p. R291S	LP	PM1, PM2, PP1, PP3, PP4
NA	NA	LGG	*NOP10*	15	NM_018648.3	c.6_7delinsC	p.L3fs	VUS-P	PVS1_moderate PM2
NA	NA	BRCA (1)	*ACD*	16	NM_001082486.2	c.1205G>A	p.W402*	LP	PVS1_verystrong , PM2
1	None	NA	*ACD*	16	NM_001082486.2	c.1213C>A	p.P405T	VUS-P (modifier)	PM2_supporting, PM3, PP4_supporting, BP4_supporting
3	None	NA	*ACD*	16	NM_001082486.2	c.250_252delAAG	p.K84del	LP	PS3_moderate PS1_supporting PS4_supporting PM2 PM4 PP4
NA	NA	MESO (1)	*ACD*	16	NM_001082486.2	c.316C>T	p.Q106*	LP	PVS1, PM2
NA	NA	GBM (1)	*ACD*	16	NM_001082486.2	c.413+1G>C	Splice site	LP	PVS1, PM2
NA	NA	COAD (1)	*ACD*	16	NM_001082486.2	c.482C>A	p.S161*	LP	PVS1, PM2
NA	NA	LIHC (1)	*ACD*	16	NM_001082486.2	c.608_610delinsG	p.P203fs	LP	PVS1, PM2
NA	NA	PCPG (1)	*ACD*	16	NM_001082486.2	c.93delinsAGCTG	p.L32fs	LP	PVS1
2	None	NA	*PARN*	16	NM_002582.4	-	-	NA	deletion encompassing PARN locus per SNP array: chr16:14,037,911 - 15,319,123
2	None	NA	*PARN*	16	NM_002582.4	c.19A>C	p.N7H	LP	PS3_supporting, PS4_supporting, PM2, PM3_supporting, PP4

3	None	NA	*PARN*	16	NM_002582.4	c.260C>T	p.S87L	LP	PS3_supporting, PM1, PM2, PP4
1	None	NA	*PARN*	16	NM_002582.4	c.-63C>T	-	VUS-P	PM2, PM3, PP4
2	None	NA	*PARN*	16	NM_002582.4	c.709C>T	p.R237*	P	PVS1, PS1_moderate, PS4_moderate, PM2, PP4
NA	NA	BRCA (1)	*PARN*	16	NM_002582.4	c.781C>T	p.Q261*	P	PVS1_very strong, PS1_supportive, PM2_moderate
1	None	LUAD (1)	*CTC1*	17	NM_025099.6	c.1186C>T	p.R396*	P	PVS1, PS1_supporting, PM2, PP4
2	None	NA	*CTC1*	17	NM_025099.6	c.1270T>G	p.C424G	LP	PM2, PM3, PP1, PP3, PP4
NA	NA	THYM (1)	*CTC1*	17	NM_025099.6	c.19C>T	p.Q7*	P	PVS1, PS1_moderate, PS4_supporting, PM2
NA	NA	GBM (1)	*CTC1*	17	NM_025099.6	c.2244delinsTC	p.G751fs	LP	PSV1, PM2
NA	NA	BLCA (1)	*CTC1*	17	NM_025099.6	c.2452C>T	p.R818*	P	PVS1, PS1_supporting. PM2
NA	NA	PRAD (1)	*CTC1*	17	NM_025099.6	c.252delinsGCCAC	p.H84fs	P	PSV1, PS1_moderate, PM2
NA	NA	BRCA (1), COAD (1)	*CTC1*	17	NM_025099.6	c.2713_2714delinsG	p.R905fs	LP	PSV1, PM2
NA	NA	MESO (1)	*CTC1*	17	NM_025099.6	c.2758+1G>T	Splice site	P	PSV1, PS1_ moderate, PM2
NA	NA	BRCA (1), SKCM (1) , THCA (1)	*CTC1*	17	NM_025099.6	c.2831_2832delinsT	p.P944fs	P	PSV1, PS1_ moderate, PS3_supporting, PS4, PM2
4	None	NA	*CTC1*	17	NM_025099.6	c.2954_2956del	p.C985del	P	PS1_moderate, PS3_supporting,

									PM2, PM3, PM4, PP4
2	None	ACC (1)	*CTC1*	17	NM_025099.6	c.2959C>T	p.R987W	LP	PS3_supporting, PS1_moderate, PM2_supporting, PM3, PP1, PP3, PP4
NA	NA	READ (1), THCA (1)	*CTC1*	17	NM_025099.6	c.3019_3020delinsT	p.L1007fs	P	PVS1, PS1_moderate, PS3_supporting, PS4_supporting
3	None	BRCA (1), CHOL (1), COAD (1), LIHC (1), LUAD (1), STAD (1)	*CTC1*	17	NM_025099.6	c.724_727del	p.K242fs	P	PVS1, PS1_moderate PS4, PS3_supporting, PM2_supporting, PM3, PP1, PP4,
NA	NA	OV (1)	*WRAP53*	17	NM_001143992.2	c.1046_1047delinsT	p.G350fs	VUS-P	PVS1, PM2_supporting
1	None	NA	*WRAP53*	17	NM_001143992.2	c.1126C>T	p.H376Y	LP	PS3_moderate, PM2, PM3, PP4
1	None	NA	*WRAP53*	17	NM_001143992.2	c.1135G>A	p.G379S	LP	PM2, PM3, PP3, PP4
1	HNSCC (1)	COAD (1), HNSCC (1)	*WRAP53*	17	NM_001143992.2	c.1192C>T	p.R398W	LP	PS3_moderate PM2_supporting PM3 PP3 PP4
1	None	NA	*WRAP53*	17	NM_001143992.2	c.1303G>A	p.G435R	LP	PS3_moderate, PM2, PM3, PP3, PP4
NA	NA	SARC (1)	*WRAP53*	17	NM_001143992.2	c.16_18delinsA	p.Q7fs	LP	PVS1, PM2_supporting
1	None	NA	*WRAP53*	17	NM_001143992.2	c.438G>A	p.W146*	LP	PVS1_moderate, PM2, PP4
1	None	NA	*WRAP53*	17	NM_001143992.2	c.492C>A	p.F164L	LP	PS1_supporting, PS3_moderate, PM2, PM3, PP4, BP4_supporting

NA	NA	BRCA (1)	*WRAP53*	17	NM_001143992.2	c.681_682delinsT	p.D228fs	LP	PVS1, PM2
4	HNSCC (post-HCT, 1)[c]	NA	*RTEL1*	20	NM_001283009.2	c.1266+3A>G	splice site	LP	PM2, PP4, PP1, PM3
2	None	NA	*RTEL1*	20	NM_001283009.2	c.1675T>A	p.F559I	LP	PS4_supporting, PM1_supporting, PM2, PM3, PP3, PP4
6	None	NA	*RTEL1*	20	NM_001283009.2	c.1861G>A	p.A621T	LP	PM1_supporting, PM2, PP1, PP3, PP4
3	HNSCC (post-HCT, 1)[c]	NA	*RTEL1*	20	NM_001283009.2	c.3289del	p.A1097fs	P	PVS1, PM2, PM3, PP1, PP4
3	None	NA	*RTEL1*	20	NM_001283009.2	c.3445del	p.Q1149fs	LP	PVS1_strong, PM2, PM3, PP1, PP4
1	None	NA	*RTEL1*	20	NM_001283009.2	c.1274T>C	p.I425T	LP	PS4_supporting, PM2, PM3, PP3, PP4
2	AML (1)	NA	*RTEL1*	20	NM_001283009.2	c.137C>T	p.T46I	LP	PM1_supporting, PM2, PM3, PP3, PP4
6	None	NA	*RTEL1*	20	NM_001283009.2	c.1476G>T	p.M492I	LP	PS1_moderate, PS3_moderate, PS4_moderate, PM2_supporting, PP1, PP3, PP4
1	None	NA	*RTEL1*	20	NM_001283009.2	c.1552A>T	p.R518*	P	PVS1, PM2, PP4
3	None	NA	*RTEL1*	20	NM_001283009.2	c.1773G>T	p.E591D	LP	PM1_supporting, PM2, PM3, PP3, PP4
1	none	NA	*RTEL1*	20	NM_001283009.2	c.2025+4A>C	splice site	VUS-P	PM2, PM3_supporting, PP3, PP4

3	BRCA (1), esophagus carcinoma (1)	NA	*RTEL1*	20	NM_001283009.2	c.2142-7C>G	splice site	LP	PS3_moderate, PM2, PM3_supporting, PP4
1	none	NA	*RTEL1*	20	NM_001283009.2	c.2387del	p.V796fs	P	PVS1, PS1_supporting, PS4_supporting, PM2, PP4
NA	NA	STAD (1)	*RTEL1*	20	NM_001283009.2	c.2869C>T	p.R957W	LP	PS4_moderate, PM2_supporting, PM3, PP4_supporting
6	none	PAAD (1)	*RTEL1*	20	NM_001283009.2	c.2920C>T	p.R974*	P	PVS1, PS1_moderate, PS3_moderate, PS4_moderate, PM2_supporting, PM3, PP1_moderate, PP4_supporting
9	none	BLCA (1)	*RTEL1*	20	NM_001283009.2	c.2956C>T	p.R986*	P	PVS1, PS1_moderate, PS4_moderate, PM2_supporting, PP1, PP4
1	none	NA	*RTEL1*	20	NM_001283009.2	c.3506C>A	p.S1169*	LP	PVS1, PM2
4	none	UCEC (1)	*RTEL1*	20	NM_001283009.2	c.3791G>A	p.R1264H	LP	PS1_moderate, PS3_moderate, PS4_supporting, PM2_supporting, PM3, PP1, PP4
3	none	NA	*RTEL1*	20	NM_001283009.2	c.49C>T	p.P17S	LP	PS1_supporting, PM1_supporting, PM2, PM3, PP1, PP3, PP4
1	none	NA	*DKC1*	X	NM_001363.5	c.103_105del	p.E35del	LP	PM1, PM2, PM4, PP4

4	HNSCC (post-HCT, 1), squamous cell ESCA and rectum carcinoid/adenocarcinoma (1, occurring subsequently)	NA	*DKC1*	X	NM_001363.5	c.1058C>T	p.A353V	LP	PS1_moderate, PS3_moderate, PS4_moderate, PM1, PP2, PP3, PP4
3	ESCA (1)	NA	*DKC1*	X	NM_001363.5	c.109_111del	p.L37del	LP	PS1_moderate, PS3_supporting, PS4_supporting, PM1, PM2, PM4, PP2, PP3, PP4
1	none	NA	*DKC1*	X	NM_001363.5	c.1168A>C	p. K390Q	LP	PS3_supporting PM2, PP2, PP3, PP4
1	none	NA	*DKC1*	X	NM_001363.5	c.1178T>A	p.I393N	LP	PM1_supporting, PM2, PP2, PP3, PP4
4	HNSCC (1)	NA	*DKC1*	X	NM_001363.5	c.1223C>T	p.T408I	LP	PS4_supporting, PM2, PP2, PP3, PP4
1	none	NA	*DKC1*	X	NM_001363.5	c.1345C>G	p.R449G	LP	PS1_supporting, PM2, PP2, PP3, PP4
1	none	NA	*DKC1*	X	NM_001363.5	c.146C>T	p.T49M	P	PS1_moderate, PS3_supporting, PS4_moderate, PM1, PM2, PM6_supporting, PP2, PP3, PP4
3	HNSCC (1)	NA	*DKC1*	X	NM_001363.5	c.160C>G	p.L54V	LP	PS3_supporting, PS4_supporting, PM2, PM1, PP1, PP2, PP3, PP4

1	none	NA	*DKC1*	X	NM_001363.5	c.191T>G	p.V64G	LP	PM1, PM2, PP2, PP3, PP4
1	none	NA	*DKC1*	X	NM_001363.5	c.196A>G	p.T66A	LP	PS1_supporting, PS3_Supporting, PM1, PM2, PP2, PP3, PP4
1	none	NA	*DKC1*	X	NM_001363.5	c.209C>T	p.T70I	LP	PS3_supporting, PM1, PM2, PP2, PP3, PP4
1	none	NA	*DKC1*	X	NM_001363.5	c.277A>T	p.N93Y	VUS-P	PM2, PP2, PP3, PP4
1	READ (1)	NA	*DKC1*	X	NM_001363.5	c.5C>T	p.A2V	LP	PS4_moderate, PM1, PM2, PS1_supporting, PP2, PP3, PP4
3	none	NA	*DKC1*	X	NM_001363.5	c.941A>G	p. K314R	LP	PS3_supporting, PS4_supporting, PM1, PM2, PP1, PP2, PP3, PP4
2	none	NA	*DKC1*	X	NM_001363.5	c.949C>T	p. L317F	LP	PS4_moderate, PM1, PM2, PP2, PP3, PP4
2	AML (1)	NA	*DKC1*	X	NM_001363.5	c.965G>A	p.R322Q	LP	PS4_supporting, PM1, PM2, PP1, PP2, PP3, PP4

[a]in part listed in Niewisch et al, Blood 2022[4]. **[b]**modified ACMG/AMP criteria as described previously in Niewisch et al, Blood 2022[4]. If not otherwise indicated cancer cases refer to occurrences without prior HCT, lung or liver transplant. If occurrence only after transplant – highlighted with "post-transplant".[c]Individual with 2 compound heterozygous RTEL1 germline variants. Therefore same case of post-transplant HNSCC.

Cancer entity abbreviations: ACC, adrenocortical carcinoma; BLCA, bladder urothelial carcinoma; BRCA, breast invasive carcinoma; CESC, cervical squamous cell carcinoma and endocervical adenocarcinoma; CHOL, cholangiocarcinoma; COAD, colon adenocarcinoma; DLBC, lymphoid neoplasm diffuse large B-cell lymphoma; ESCA, esophageal carcinoma; GBM, glioblastoma multiforme; HNSCC, head and neck squamous cell carcinoma; KICH, kidney chromophobe; KIRC, kidney renal clear cell carcinoma; KIRP, kidney renal papillary cell carcinoma; LAML, acute myeloid leukemia; LGG, brain lower graded glioma; LIHC, liver hepatocellular carcinoma; LUAD, lung adenocarcinoma; LUSC, lung squamous cell carcinoma; MESO, mesothelioma; MDS, myelodysplastic syndrome; OV, ovarian serous cystadenocarcinoma; PAAD, pancreatic adenocarcinoma; PCPG, pheochromocytoma and paraganglioma; PRAD, prostate adenocarcinoma; READ, rectum adenocarcinoma; SARC, sarcoma; SKCM, skin cutaneous melanoma; TGCT, testicular germ cell tumors; THCA, thyroid carcinoma; THYM, thymoma; UCEC, uterine carcinosarcoma; UCS, uterine corpus endometrial carcinoma; UVM, uveal melanoma.

<u>eTable 6:</u> National Cancer Institute's elomer Biology isorder Cohort pretransplant cancer data comparison with Surveillance, Epidemiology, and End Results (SEER) Program. 230 Subjects followed from birth to transplant (hematopoietic cell and/or lung and/or liver transplant) or last contact (7697.59 person years).

Cancer Event	Observed	Expected	O/E	CI Lower	CI Upper	Excess Risk
All Sites	34	10.19+	3.34	2.31	4.66	30.93
All Sites excluding Non-Melanoma Skin	34	10.14+	3.35	2.32	4.68	30.99
All Solid Tumors and Lymphoma	28	9.42+	2.97	1.97	4.29	24.13
All Solid Tumors	23	8.76+	2.63	1.66	3.94	18.5
Oral Cavity and Pharynx (All)	14	0.26+	54.37	29.72	91.22	17.85
Tongue	12	0.08+	158.50	81.9	276.87	15.49
Gum and Other Mouth	1	0.03+	30.86	0.78	171.92	1.26
Other Oral Cavity and Pharynx	1	0.00+	229.05	5.8	1276.19	1.29
Digestive System (All)	6	1.35+	4.45	1.63	9.69	6.04
Esophagus	3	0.07+	44.24	9.12	129.28	3.81
Rectum	2	0.19+	10.63	1.29	38.39	2.35
Anus, Anal Canal and Anorectum	1	0.04+	24.08	0.61	134.19	1.25
Breast	1	1.86+	0.54	0.01	3	-1.11
Female Genital System (All)	2	0.80+	2.49	0.3	9.01	1.56
Cervix Uteri	1	0.19+	5.31	0.13	29.59	1.05
Corpus Uteri	1	0.36+	2.8	0.07	15.62	0.84
All Lymphatic and Hematopoietic Diseases (All)	11	1.17+	9.37	4.68	16.76	12.76
Lymphoma (All)	5	0.66+	7.52	2.44	17.55	5.63
Non-Hodgkin Lymphoma (All)	5	0.46+	10.80	3.51	25.19	5.89
• NHL - Nodal	3	0.32+	9.51	1.96	27.78	3.49
• NHL - Extranodal	2	0.15+	13.55	1.64	48.96	2.41
Leukemia (All)	6	0.42+	14.28	5.24	31.09	7.25
Acute Myeloid Leukemia	5	0.10+	49.50	16.07	115.51	6.36
Other Acute Leukemia	1	0.01+	108.34	2.74	603.62	1.29

Excess risk is per 10,000; Confidence intervals are 95%. + = the required age or year was not found in the referent rate table; therefore the closest age/year was used to obtain the rate.

__eTable 7__**: National Cancer Institute’s Telomer Biology Disorder Cohort pretransplant cancer data comparison with Surveillance, Epidemiology, and End Results (SEER) Program for the autosomal dominant subgroup.** 139 Subjects followed from birth to transplant (hematopoietic cell and/or lung and/or liver transplant) or last contact (5750.5 person years).

Event	Observed	Expected	O/E	CI Lower	CI Upper	Excess Risk
All Sites	23	9.03+	2.55	1.62	3.82	24.3
All Sites excluding Non-Melanoma Skin	23	8.99+	2.56	1.62	3.84	24.37
All Solid Tumors and Lymphoma	18	8.40+	2.14	1.27	3.39	16.7
All Solid Tumors	14	7.84+	1.78	0.98	2.99	10.7
Oral Cavity and Pharynx (All)	10	0.23+	43.12	20.68	79.29	16.99
Tongue	10	0.07+	145.41	69.73	267.42	17.27
Digestive System (All)	1	1.22+	0.82	0.02	4.58	-0.37
Esophagus	1	0.06+	15.89	0.4	88.51	1.63
Breast	1	1.70+	0.59	0.01	3.27	-1.22
Female Genital System (All)	2	0.74+	2.7	0.33	9.76	2.19
Cervix Uteri	1	0.18+	5.62	0.14	31.31	1.43
Corpus Uteri	1	0.33+	3.05	0.08	16.99	1.17
All Lymphatic and Hematopoietic Diseases	9	0.96+	9.41	4.3	17.86	13.99
Lymphoma (All)	4	0.55+	7.27	1.98	18.61	6
Non-Hodgkin Lymphoma	4	0.39+	10.19	2.78	26.1	6.27
• NHL - Nodal	2	0.27+	7.46	0.9	26.94	3.01
• NHL - Extranodal	2	0.12+	16.10	1.95	58.15	3.26
Leukemia (All)	5	0.32+	15.39	5	35.91	8.13
Acute Myeloid Leukemia	4	0.08+	48.49	13.21	124.14	6.81
Other Acute Leukemia	1	0.01+	139.97	3.54	779.84	1.73

Excess risk is per 10,000; Confidence intervals are 95%. + = the required age or year was not found in the referent rate table, therefore the closest age/year was used to obtain the rate.

<u>eTable 8:</u> National Cancer Institute's Telomer Biology Disorder Cohort pretransplant cancer data comparison with Surveillance, Epidemiology, and End Results (SEER) Program for the autosomal recessive/X-linked subgroup. 64 subjects followed from birth to transplant (hematopoietic cell and/or lung and/or liver transplant) or last contact (1377.42 person years). CI, 95% confidence interval; O/E, observed/expected.

Event	Observed	Expected	O/E	CI Lower	CI Upper	Excess Risk
All Sites	10	0.53+	19.03	9.12	34.99	68.78
All Sites excluding Non-Melanoma Skin	10	0.52+	19.16	9.19	35.24	68.81
All Solid Tumors and Lymphoma	9	0.45+	20.16	9.22	38.28	62.1
All Solid Tumors	9	0.38+	23.97	10.96	45.5	62.61
Oral Cavity and Pharynx (All)	4	0.01+	276.00	75.2	706.67	28.93
Tongue	2	0.00+	496.66	60.15	1794.1	14.49
Gum and Other Mouth	1	0.00+	668.10	16.91	3,722.43	7.25
Other Oral Cavity and Pharynx	1	0.00+	5,212.81	131.98	29043.92	7.26
Digestive System (All)	5	0.05+	94.97	30.84	221.64	35.92
Esophagus	2	0.00+	819.78	99.28	2961.34	14.5
Rectum	2	0.01+	236.58	28.65	854.61	14.46
Anus, Anal Canal and Anorectum	1	0.00+	676.75	17.13	3770.59	7.25
All Lymphatic and Hematopoietic Diseases	1	0.14+	7.34	0.19	40.92	6.27
Acute Myeloid Leukemia	1	0.01+	88.64	2.24	493.87	7.18

Excess risk is per 10,000; Confidence intervals are 95%. + = the required age or year was not found in the referent rate table, therefore the closest age/year was used to obtain the rate.

<u>eTable 9:</u> National Cancer Institute's Telomer Biology Disorder Cohort pretransplant cancer data comparison with Surveillance, Epidemiology, and End Results (SEER) Program for the *TINF2* subgroup. 27 subjects followed from birth to transplant (hematopoietic cell and/or lung and/or liver transplant) or last contact (569.67 person years).

Event	Observed	Expected	O/E	CI Lower	CI Upper	Excess Risk
All Sites	1	0.64+	1.57	0.04	8.73	6.36
All Sites excluding Non-Melanoma Skin	1	0.64+	1.57	0.04	8.77	6.41
All Solid Tumors and Lymphoma	1	0.58+	1.72	0.04	9.59	7.36
All Lymphatic and Hematopoietic Diseases (All)	1	0.08+	12.26	0.31	68.33	16.12
Lymphoma (All)	1	0.04+	23	0.58	128.17	16.79
Non-Hodgkin Lymphoma	1	0.03+	33.25	0.84	185.27	17.03
• NHL - Nodal	1	0.02+	48.53	1.23	270.38	17.19

Excess risk is per 10,000; Confidence intervals are 95%. + = the required age or year was not found in the referent rate table; therefore the closest age/year was used to obtain the rate.

<u>eTable 10:</u> National Cancer Institute's Telomer Ciology Disorder Cohort post-transplant cancer data comparison with Surveillance, Epidemiology, and End Results (SEER) Program. 74 Subjects followed from transplant or last contact (338.26 person years at risk). There were four people with zero person time (transplant = last contact)

Event	Observed	Expected	O/E	CI Lower	CI Upper	Excess Risk
All Sites	13	0.52+	24.98	13.3	42.72	368.93
All Sites excluding Non-Melanoma Skin	13	0.52+	25.08	13.35	42.89	369
All Solid Tumors and Lymphoma	12	0.48+	25.00	12.92	43.67	340.57
All Solid Tumors	11	0.45+	24.57	12.26	43.96	311.96
Oral Cavity and Pharynx (All)	8	0.02+	483.88	208.91	953.44	236.02
Tongue	3	0.01+	519.95	107.23	1519.52	88.52
Gum and Other Mouth	5	0.00+	3,063.69	994.77	7,149.64	147.77
Skin excluding Basal and Squamous	2	0.04+	46.64	5.65	168.47	57.86
Melanoma of the Skin	2	0.04+	49.06	5.94	177.21	57.92
Urinary System (All)	1	0.03+	30.17	0.76	168.08	28.58
Urinary Bladder	1	0.01+	70.41	1.78	392.3	29.14
All Lymphatic and Hematopoietic Diseases	2	0.06+	33.91	4.11	122.49	57.38
Lymphoma (All)	1	0.03+	30.99	0.78	172.68	28.61
Hodgkin Lymphoma	1	0.01+	119.61	3.03	666.42	29.32
• Hodgkin - Nodal	1	0.01+	121.72	3.08	678.16	29.32
Leukemia (All)	1	0.02+	46.94	1.19	261.54	28.93
Acute Myeloid Leukemia	1	0.01+	196.93	4.99	1,097.20	29.41

Excess risk is per 10,000; Confidence intervals are 95%. + = the required age or year was not found in the referent rate table, therefore the closest age/year was used to obtain the rate.

eTable 11: **National Cancer Institute's Telomer Biology Disorder Cohort post-transplant cancer data comparison with Surveillance, Epidemiology, and End Results (SEER) Program for the autosomal dominant subgroup.**

28 Subjects followed from transplant or last contact (100.34 person years at risk)

Event	Observed	Expected	O/E	CI Lower	CI Upper	Excess Risk
All Sites	3	0.43+	6.95	1.43	20.32	255.99
All Sites excluding Non-Melanoma Skin	3	0.43+	6.98	1.44	20.39	256.14
All Solid Tumors and Lymphoma	2	0.40+	4.96	0.6	17.91	159.12
All Solid Tumors	2	0.38+	5.23	0.63	18.9	161.22
Skin excluding Basal and Squamous	2	0.03+	60.26	7.3	217.69	196.01
Melanoma of the Skin	2	0.03+	63.13	7.65	228.06	196.16
All Lymphatic and Hematopoietic Diseases	1	0.04+	26.18	0.66	145.87	95.85
Leukemia (All)	1	0.01+	84.01	2.13	468.06	98.47
Acute Myeloid Leukemia	1	0.00+	320.01	8.1	1,782.99	99.35

Excess risk is per 10,000; Confidence intervals are 95%. + = the required age or year was not found in the referent rate table, therefore the closest age/year was used to obtain the rate.

eTable 12: National Cancer Institute's Telomer Biology Disorder Cohort post-transplant cancer data comparison with Surveillance, Epidemiology, and End Results (SEER) Program for the autosomal recessive/X-linked subgroup. 29 Subjects followed from transplant or last contact (137.34 person years at risk)

Event	Observed	Expected	O/E	CI Lower	CI Upper	Excess Risk
All Sites	7	0.05+	135.08	54.31	278.31	505.91
All Sites excluding Non-Melanoma Skin	7	0.05+	136.11	54.72	280.44	505.94
All Solid Tumors and Lymphoma	7	0.04+	156.36	62.87	322.17	506.43
All Solid Tumors	6	0.04+	162.06	59.47	352.74	434.18
Oral Cavity and Pharynx (All)	6	0.00+	6,475.78	2,376.5	14,095.04	436.81
Tongue	1	0.00+	3,586.17	90.79	19,980.84	72.79
Gum and Other Mouth	5	0.00+	42,115.05	13674.6	98,282.47	364.05
All Lymphatic and Hematopoietic Diseases	1	0.01+	75.61	1.91	421.27	71.85
Lymphoma (All)	1	0.01+	129.13	3.27	719.44	72.25
Hodgkin Lymphoma	1	0.00+	253.81	6.43	1414.11	72.53
• Hodgkin Lymphoma - Nodal	1	0.00+	257.16	6.51	1432.81	72.53

Excess risk is per 10,000; Confidence intervals are 95%. + = the required age or year was not found in the referent rate table; therefore the closest age/year was used to obtain the rate.

<u>eTable 13:</u> National Cancer Institute's Telomer Biology Disorder Cohort post-transplant cancer data comparison with Surveillance, Epidemiology, and End Results (SEER) Program for the *TINF2* subgroup. 17 Subjects followed from transplant or last contact (100.58 person years at risk)

Event	Observed	Expected	O/E	CI Lower	CI Upper	Excess Risk
All Sites	3	0.04+	80.61	16.62	235.57	294.57
All Sites excluding Non-Melanoma Skin	3	0.04+	81.07	16.72	236.92	294.59
All Solid Tumors and Lymphoma	3	0.03+	93.98	19.38	274.65	295.1
All Solid Tumors	3	0.03+	105.29	21.71	307.7	295.44
Oral Cavity and Pharynx (All)	2	0.00+	3,702.91	448.44	13,376.19	198.79
Tongue	2	0.00+	12,632.48	1,529.85	45,632.85	198.83
Urinary System (All)	1	0.00+	828.41	20.97	4,615.61	99.3
Urinary Bladder	1	0.00+	4,752.66	120.33	26,480.1	99.4

Excess risk is per 10,000; Confidence intervals are 95%. + = the required age or year was not found in the referent rate table; therefore the closest age/year was used to obtain the rate.

<u>eTable 14:</u> National Cancer Institute's Telomer Biology Disorder Carrier Cohort cancer data comparison with Surveillance, Epidemiology, and End Results (SEER) Program. Subjects with monoallelic germline variants in *WRAP53* or *CTC1* followed from birth to last contact (2139.63 person years)

Event	Observed	Expected	O/E	CI Lower	CI Upper	Excess Risk
All Sites	1	4.82+	0.21	0.01	1.16	-17.88
All Sites excluding Non-Melanoma Skin	1	4.80+	0.21	0.01	1.16	-17.79
All Solid Tumors and Lymphoma	1	4.52+	0.22	0.01	1.23	-16.48
All Solid Tumors	1	4.28+	0.23	0.01	1.3	-15.34
Breast	1	1.34+	0.75	0.02	4.17	-1.58

Excess risk is per 10,000; Confidence intervals are 95%. + = the required age or year was not found in the referent rate table; therefore the closest age/year was used to obtain the rate.

<u>eTable 15:</u> The Cancer Genome Atlas - characteristics of cancer cases with pathogenic/likely pathogenic in Telomere Biology Disorder associated genes

**Cohort characteristics**	**The Cancer Genome Atlas**		
**Cohort subsets**	uTBD	POT1-TPD	TBD carriers
**No. of individuals**	17	10	31
**Affected genes (No. of patients harboring pathogenic variant)**	*ACD (5)*[a]<br>*TERT (3)*[b]<br>*RTEL1 (4)*<br>*TINF2 (2)*<br>*PARN (1)*<br>*NAF1 (1)*<br>*NOP10 (1)*	*POT1 (10)*[a]	*CTC1 (20)*[c]<br>*STN1 (6)*[d]<br>*WRAP53 (5)*[e]
**Sex (Male/Female)**	6/11	5/5	13/17[f]
**Race/Ethnicity**[c]			
• White	12	8	25
• Asian	1	1	0
• Black or African America	1	0	2
• Native American	0	0	0
• Mixed	0	0	0
• Unknown	3	1	4
**Median year of birth (range)**	1941<br>(1920-1988)	1954<br>(1921-1972)	1947<br>(1918-1977)[g]
**Median age at cancer diagnosis**	68.6 years (24.5-89.34)	57 years (37.1-84.9)	64.8 (33.2-83.6) [g]
**Deceased at last follow-up**	3	4	8[f]

[a] One liver hepatocellular carcinoma carried both a LOF variant in ACD (p.P203fs) and in POT1 (p.R363*) and was evaluated as POT1-TPD. Therefore**,** this case is not included and listed with the uTBDs. [b] All 3 TERT cases harbored the same variant (p.R1086H). [c] For CTC1 the most common shared variant was p.K242Lfs (n=6), followed by p.P944Lfs (n=3), p.L1007Cfs and p.R905Gfs (2 each). [d] Three STN1 cases shared the same LOF variant (p.R133X).[e] Two cases shared the same missense variant (WRAP53 p.R398W). [f]one case missing information..[g] Two cases with missing information.

Abbreviations: POT1-TPD, POT1 tumor predisposition (Heterozygous POT1 variants associated with longer telomeres and elevated cancer risk have recently been termed POT1-tumor predisposition syndrome; uTBD, unrecognized telomere biology disorder.

eTable 16: **The Cancer Genome Atlas – cancer cases with pathogenic/likely pathogenic TBD-associated variants per cancer category**

Cancer category	Included cancer entities	Total	uTBD cases with P/LP variant in AD or AD/AR TBD gene (% of total)	POT1-TPD cases with monoallelic P/LP variant in POT1 (% of total)	TBD carrier cases with P/LP variant in AR TBD gene (% of total)
**All sites**	NA	9089	17 (0.2)	10 (0.1)	31 (0.3)
**All solid tumors except lymphoma**	NA	8903	17 (0.2)	9 (0.1)	31 (0.4)
**Uveal melanoma**	UVM	80	0	1 (1.3)	0
**Endocrine**	THCA, ACC, PCPG, THYM	807	1 (0.1)	1 (0.1)	7 (0.9)
**Brain and other nervous system**	GBM, LGG	907	3 (0.3)	1 (0.1)	1 (0.1)
**Breast**	BRCA	960	2 (0.2)	1 (0.1)	4 (0.4)
**Respiratory system**	LUAD, LUSC<br>MESO	730<br>82	1 (0.1)<br>1 (1.2)	0<br>0	2 (0.3)<br>2 (2.4)
**Oral Cavity and Pharynx**	HNSCC	509	0	0	1 (0.2)
**Gastrointestinal system**	ESCA, STAD<br>CHOL, LIHC<br>PAAD<br>COAD<br>READ	522<br>369<br>153<br>392<br>153	1 (0.2)<br>0<br>1 (0.7)<br>2 (0.5)<br>0	1 (0.2)<br>1 (0.3)<br>0<br>0<br>1 (0.7)	2 (0.4)<br>2 (0.6)<br>0<br>3 (0.8)<br>1 (0.7)
**Female Genital System**	UCEC, UCS, OV<br>CESC	919<br>300	2 (0.2)<br>0	0<br>0	2 (0.2)<br>0
**Male Genital System**	PRAD, TGCT	590	1 (0.2)	1 (0.2)	1 (0.2)
**Urinary System**	KICH, KIRC, KIRP<br>BLCA	332<br>392	0<br>1 (0.3)	0<br>0	0<br>1 (0.3)
**Melanoma**	SKCM	469	1 (0.2)	0	1 (0.2)
**Sarcoma**	SARC	237	0	1 (0.4)	1 (0.4)
**Lymphatic and Hematopoietic Diseases (excluding MDS)**	AML, DLBC	186	0	1 (0.5)	0

Cancer entity abbreviations: ACC, adrenocortical carcinoma; BLCA, bladder urothelial carcinoma; BRCA, breast invasive carcinoma; CESC, cervical squamous cell carcinoma and endocervical adenocarcinoma; CHOL, cholangiocarcinoma; COAD, colon adenocarcinoma; DLBC, lymphoid neoplasm diffuse large B-cell lymphoma; ESCA, esophageal carcinoma; GBM, glioblastoma multiforme; HNSCC, head and neck squamous cell carcinoma; KICH, kidney chromophobe; KIRC, kidney renal clear cell carcinoma; KIRP, kidney renal papillary cell carcinoma; LAML, acute myeloid leukemia; LGG, brain lower graded glioma; LIHC, liver hepatocellular carcinoma; LUAD, lung adenocarcinoma; LUSC, lung squamous cell carcinoma; MESO, mesothelioma; MDS, myelodysplastic syndrome; OV, ovarian serous cystadenocarcinoma; PAAD, pancreatic adenocarcinoma; PCPG, pheochromocytoma and paraganglioma; PRAD, prostate adenocarcinoma; READ, rectum adenocarcinoma; SARC, sarcoma; SKCM, skin cutaneous melanoma; TGCT, testicular germ cell tumors; THCA, thyroid carcinoma; THYM, thymoma; UCEC, uterine carcinosarcoma; UCS, uterine corpus endometrial carcinoma; UVM, uveal melanoma.

Abbreviations: AD, autosomal dominant; autosomal recessive (AR); XLR, X-linked recessive; P/LP, pathogenic/likely pathogenic; TBD, telomere biology disorder

<u>eFigure 1</u>: Overview of study cohort selection within the National Cancer Institute's Inherited Bone Marrow Failure Syndromes Study.

The National Cancer Institute's inherited bone marrow failure (NCI IBMFS, clinicaltrials.gov NCT00027274, https://marrowfailure.cancer.gov) study enrolls affected individuals and their immediate family members who have a proven IBMFS or bone marrow failure which appears to be inherited but has no clear genetic basis. AR, autosomal recessive; TBD, telomere biology disorder.

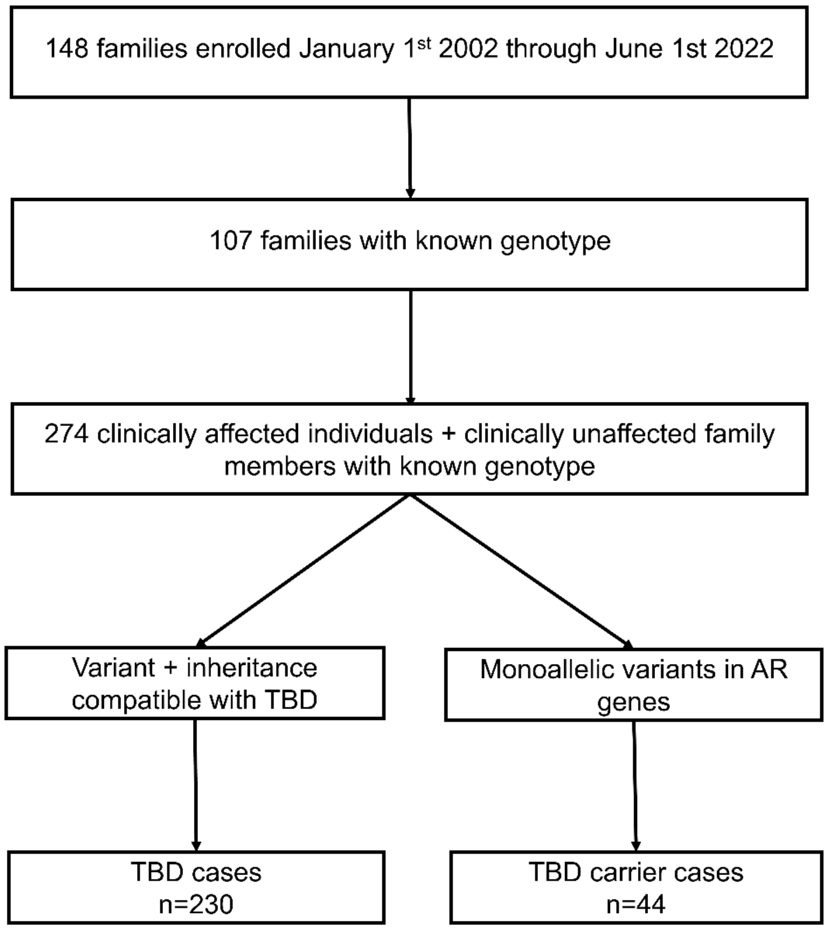

eFigure 2: Overview of germline variant curation and case selection within The Cancer Genome Atlas dataset

A. **Overview of germline variant calling, annotation and curation in The Cancer Genome Atlas dataset**

B. **Overview of study cohort selection within The Cancer Genome Atlas dataset.** *Niewisch et al. 2022[4]. Richards et al. 2015[5].

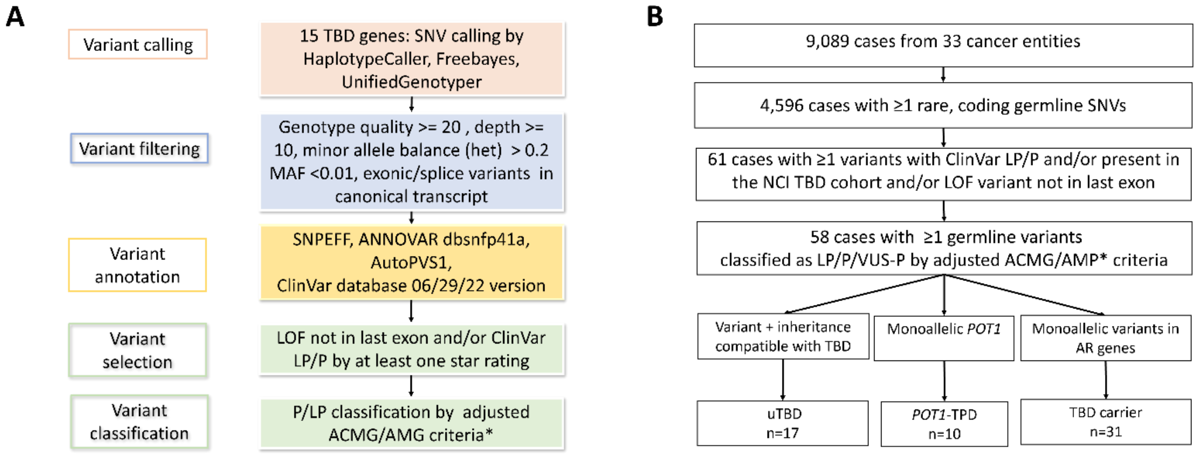

Abbreviations: ACMG/AMP, American College of Medical Genetics and Genomics and the Association for Molecular Pathology; AR, autosomal recessive; NCI, National Cancer Institute; LP, likely pathogenic; P, pathogenic; TBD carriers, monoallelic P/LP variant in a TBD-related gene solely associated with AR inheritance (*e.g., WRAP53* or *CTC1*) or female carriers of P/LP variants in *DKC1. POT1*-TPD, *POT1*-Tumor predisposition: Monoallelic POT1 variants are primarily associated with POT1 tumor predisposition (POT1-TPD) and associated with long telomeres[6]. Recently, a monoallelic POT1 variant was detected in a family with TBD and variably short telomere length, suggesting that monoallelic POT1 variants may result in either shortened or elongated telomeres[2]. Given incomplete phenotype data within the TCGA dataset, we considered AR POT1 as TBD and AD POT1 as POT1-TPD; uTBD, unrecognized Telomere Biology Disorder case defined genetically by the presence of a germline P/LP variant in a TBD gene in conjunction with an inheritance pattern consistent with a TBD; TCGA, The Cancer Genome Atlas.

<u>eFigure 3:</u> Transplant and cancer-free survival in the TBD cohort using Kaplan-Meier estimates

A. Cancer and transplant (hematopoietic cell, lung, and/or liver transplant) free survival for the complete cohort.
B. Cancer and transplant (hematopoietic cell, lung, and/or liver transplant) free survival by genotype subgroups based on mode of inheritance.

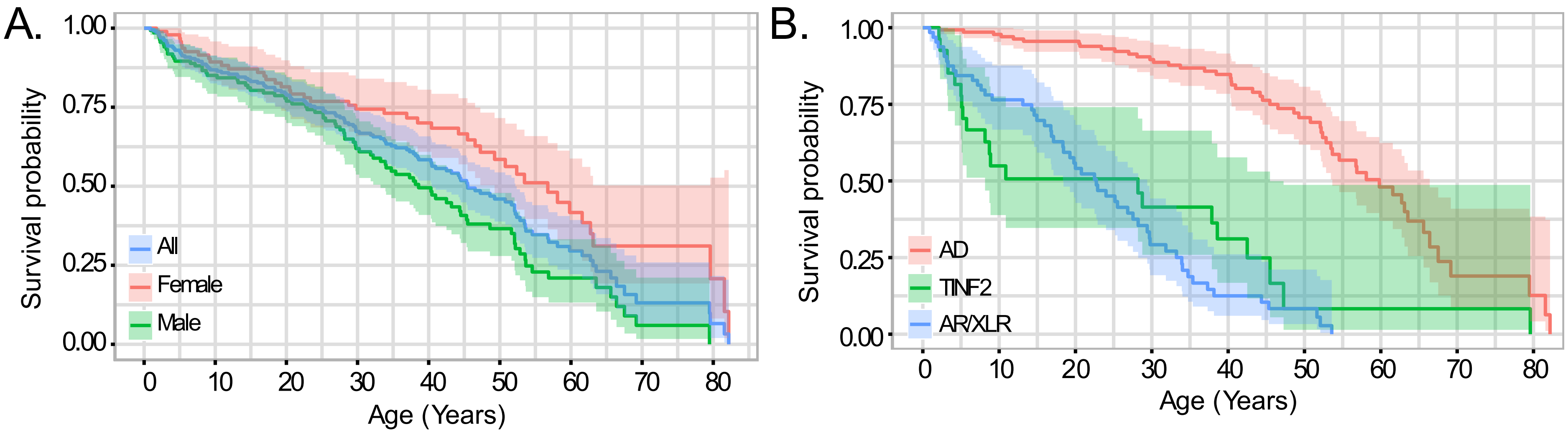

Number at risk

All	230	192	160	122	85	51	21	5	2
Female	95	81	71	60	42	26	13	3	2
Male	135	111	89	62	43	25	8	2	0

AD	139	131	117	99	74	47	20	4	2
TINF2	27	13	12	9	5	1	1	1	0
AR/XLR	64	48	31	14	6	3	0	0	0

Abbreviations: AD, autosomal dominant, non-TINF2 associated telomere biology disorders (TBDs), AR, autosomal recessive; TINF2, autosomal dominant-TINF2 associated TBDs; XLR, X-linked recessive TBDs.

<u>eFigure4:</u> Complications in unrelated, non-transplanted Telomere Biology Disorder individuals.

Cumulative incidences of adverse events by age in patients enrolled in the National Cancer Institute's Telomere Biology Disorder cohort (NCI TBD cohort). Cumulative incidence (CI) of each event as initial cause of failure (stair-step lines). Red indicates CI of hematologic malignancies; green indicates CI of solid tumors; blue indicates CI of transplant/death.

A. 107 patients with genetically proven telomere biology disorder. B. Subset of 42 patients with autosomal dominant telomere biology disorder, non*TINF2.* C. Subset of 48 patients with biallelic/X-linked disease (autosomal recessive) telomere biology disorder. D. Subset of 17 patients with autosomal dominant *TINF2* disease

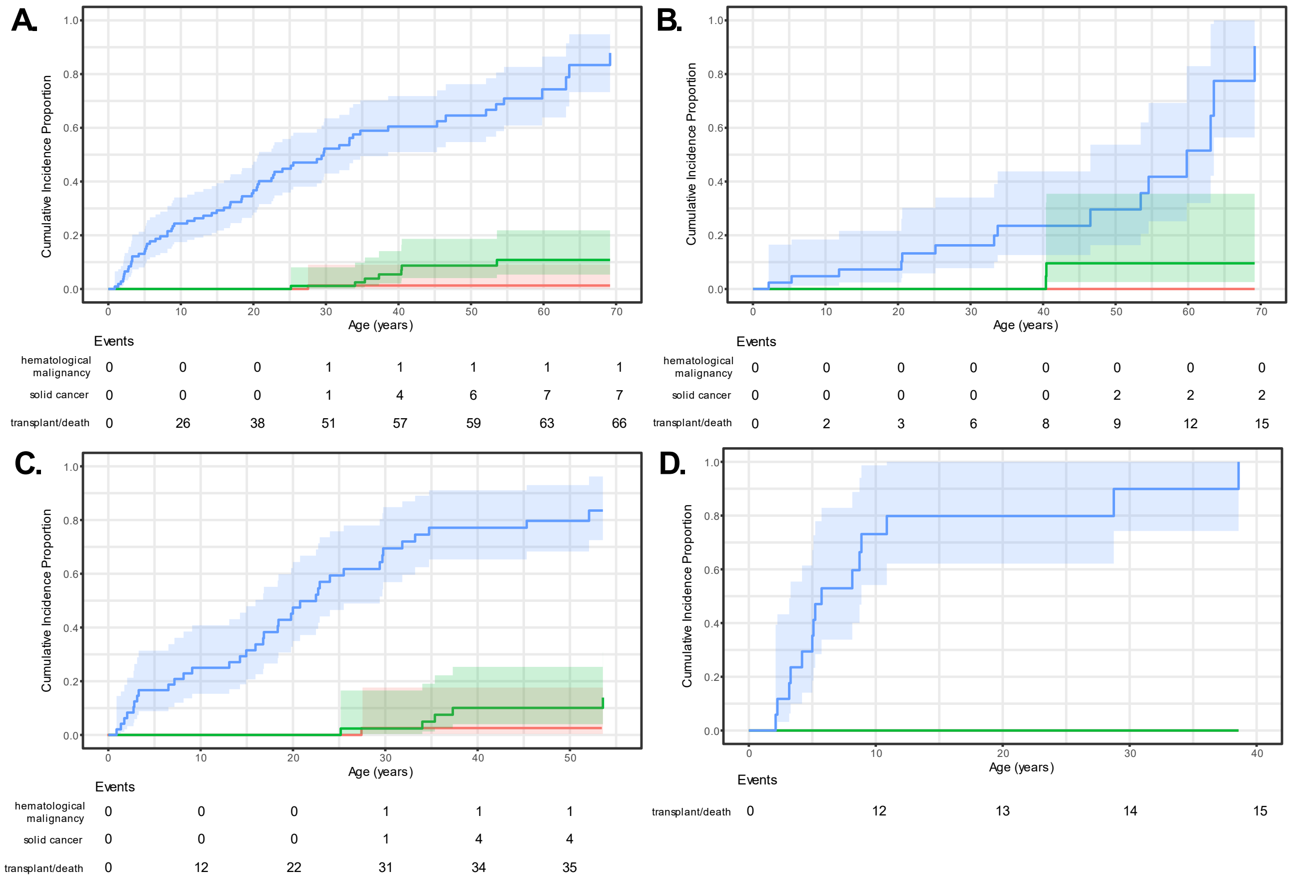

References

1. Revy P, Kannengiesser C, Bertuch AA. Genetics of human telomere biology disorders. *Nat Rev Genet*. Feb 2023;24(2):86-108. doi:10.1038/s41576-022-00527-z
2. Kelich J, Aramburu T, van der Vis JJ, et al. Telomere dysfunction implicates POT1 in patients with idiopathic pulmonary fibrosis. *J Exp Med*. May 2 2022;219(5)doi:10.1084/jem.20211681
3. Huang KL, Mashl RJ, Wu Y, et al. Pathogenic Germline Variants in 10,389 Adult Cancers. *Cell*. Apr 5 2018;173(2):355-370.e14. doi:10.1016/j.cell.2018.03.039
4. Niewisch MR, Giri N, McReynolds LJ, et al. Disease progression and clinical outcomes in telomere biology disorders. *Blood*. Mar 24 2022;139(12):1807-1819. doi:10.1182/blood.2021013523
5. Richards S, Aziz N, Bale S, et al. Standards and guidelines for the interpretation of sequence variants: a joint consensus recommendation of the American College of Medical Genetics and Genomics and the Association for Molecular Pathology. *Genet Med*. May 2015;17(5):405-24. doi:10.1038/gim.2015.30
6. Wu Y, Poulos RC, Reddel RR. Role of POT1 in Human Cancer. *Cancers (Basel)*. Sep 24 2020;12(10)doi:10.3390/cancers12102739
