# Supplementary material for: Genotype and Associated Cancer Risk in Individuals With Telomere Biology Disorders
Source: JAMA Netw Open. 2024 Dec 11;7(12):e2450111. doi: 10.1001/jamanetworkopen.2024.50111 (PMC11635530; doi:10.1001/jamanetworkopen.2024.50111)
Supplement: Supplement 2. — Data Sharing Statement [file jamanetwopen-e2450111-s002.pdf]

Data Sharing Statement

Niewisch. Genotype and Associated Cancer Risk in Individuals With Telomere Biology Disorders. *JAMA Netw Open*. Published December 11, 2024.
doi:10.1001/jamanetworkopen.2024.50111

Data
Data available: Yes
Data types: Deidentified participant data
How to access data: De-identified participant data will be made available to qualified researchers after establishment of institutional data transfer agreements. Contact Dr. Sharon Savage, savagesh@mail.nih.gov, to initiate collaborations.
When available: With publication

Supporting Documents
Document types: None

Additional Information
Who can access the data: De-identified participant data will be made available to qualified researchers after establishment of institutional data transfer agreements. Contact Dr. Sharon Savage, savagesh@mail.nih.gov, to initiate collaborations.
Types of analyses: Data will be made available for specific analyses after discussion with the requestor.
Mechanisms of data availability: De-identified participant data will be made available to qualified researchers after establishment of institutional data transfer agreements. Contact Dr. Sharon Savage, savagesh@mail.nih.gov, to initiate collaborations.
